# Links between the three-dimensional movements of whale sharks (*Rhincodon typus*) and the bio-physical environment off a coral reef

**DOI:** 10.1186/s40462-024-00452-2

**Published:** 2024-01-31

**Authors:** Ben D’Antonio, Luciana C. Ferreira, Mark Meekan, Paul G. Thomson, Lilian Lieber, Patti Virtue, Chloe Power, Charitha B. Pattiaratchi, Andrew S. Brierley, Ana M. M. Sequeira, Michele Thums

**Affiliations:** 1 Oceans Graduate School and the UWA Oceans Institute, The University of Western Australia, 35 Stirling Highway, Perth, WA 6009 Australia; 2 Australian Institute of Marine Science, Indian Ocean Marine Research Centre, University of Western Australia, Perth, WA Australia; 3 The Oceans Institute, University of Western Australia, Perth, WA Australia; 4 Marine Biological Association of the United Kingdom, The Laboratory, Citadel Hill, Plymouth, PL1 2PB UK; 5 Institute for Marine and Antarctic Studies, University of Tasmania, Hobart, TAS Australia; 6 CSIRO Environment, Battery Point, TAS 7004 Australia; 7 Pelagic Ecology Research Group, Scottish Oceans Institute, Gatty Marine Laboratory, School of Biology, University of St. Andrews, St Andrews, KY16 8LB Scotland, UK; 8 Research School of Biology, Division of Ecology and Evolution, The Australian National University, 46 Sullivans Creek Road, Canberra, ACT 2600 Australia

**Keywords:** Marine megafauna, Predator–prey, Foraging ecology, Bio-physical drivers, Zooplankton, Habitat use, Oceanography, 3D utilisation distribution

## Abstract

**Background:**

Measuring coastal-pelagic prey fields at scales relevant to the movements of marine predators is challenging due to the dynamic and ephemeral nature of these environments. Whale sharks (*Rhincodon typus*) are thought to aggregate in nearshore tropical waters due to seasonally enhanced foraging opportunities. This implies that the three-dimensional movements of these animals may be associated with bio-physical properties that enhance prey availability. To date, few studies have tested this hypothesis.

**Methods:**

Here, we conducted ship-based acoustic surveys, net tows and water column profiling (salinity, temperature, chlorophyll fluorescence) to determine the volumetric density, distribution and community composition of mesozooplankton (predominantly euphausiids and copepods) and oceanographic properties of the water column in the vicinity of whale sharks that were tracked simultaneously using satellite-linked tags at Ningaloo Reef, Western Australia. Generalised linear mixed effect models were used to explore relationships between the 3-dimensional movement behaviours of tracked sharks and surrounding prey fields at a spatial scale of ~ 1 km.

**Results:**

We identified prey density as a significant driver of horizontal space use, with sharks occupying areas along the reef edge where densities were highest. These areas were characterised by complex bathymetry such as reef gutters and pinnacles. Temperature and salinity profiles revealed a well-mixed water column above the height of the bathymetry (top 40 m of the water column). Regions of stronger stratification were associated with reef gutters and pinnacles that concentrated prey near the seabed, and entrained productivity at local scales (~ 1 km). We found no quantitative relationship between the depth use of sharks and vertical distributions of horizontally averaged prey density. Whale sharks repeatedly dove to depths where spatially averaged prey concentration was highest but did not extend the time spent at these depth layers.

**Conclusions:**

Our work reveals previously unrecognized complexity in interactions between whale sharks and their zooplankton prey.

**Supplementary Information:**

The online version contains supplementary material available at 10.1186/s40462-024-00452-2.

## Background

Movement facilitates a range of ecological processes that cascade through communities and ecosystems [1]. Consequently, understanding how and why animals move is a central question in ecology [2]. For predators, movement has been attributed to a range of intrinsic (e.g., reproductive status, sex and age; [3]) and extrinsic factors (e.g., temperature or shift in prey distribution; [4, 5]). In marine environments, the detectability and accessibility of prey can vary with changing bio-physical conditions [6–8], forcing predators to track prey distributions, or ‘prey fields’, that are patchy, ephemeral, and hierarchical in structure across different spatial and temporal scales [9, 10]. To feed successfully, marine predators are thus expected to employ movement strategies that maximise prey encounter rates [11, 12], and modify foraging behaviours to enhance prey accessibility and consumption [13–15]. Although challenging, understanding the influence of prey-field characteristics on the movement patterns and behaviours of marine predators is fundamental to our understanding of their ecology and to predict how they may respond to environmental change [16].

Acoustic surveys enable the quantification of pelagic prey-field metrics (e.g., prey field depth and density) at spatial and temporal resolutions comparable to the movement patterns and behaviour of marine predators [17, 18]. Such methods have enabled a number of studies to describe the movement patterns of air-breathing marine predators (e.g., pinnipeds, rorqual whales and penguins) in relation to surrounding prey fields across meso- scales (10–100 s km; [19, 20]), sub-mesoscale (1–10 km; [15]) or local (meters) spatial scales [21]. For example, rorqual whales have been shown to alter their diving behaviour [22] and concentrate foraging efforts [15, 23] in relation to prey densities and distribution. However, relatively few studies have combined concurrent measurements of prey density with the movements of large gill-breathing and highly mobile predators such as sharks [24].

The whale shark (*Rhincodon typus*) is the world’s largest shark (up to 18 m total length; [25]) and, similar to rorqual whales, feeds on prey many orders of magnitude smaller (< 1 g) than their own body mass. This means that whale sharks must regularly consume enormous quantities of prey to sustain their energetic demands [26, 27]. This is a challenge that is exacerbated by the warm temperatures (that drive high metabolic rates) and oligotrophic conditions (limited prey availability) of the tropical waters in which they reside. For this reason, whale sharks form relatively predictable aggregations in tropical coastal waters in response to seasonally enhanced foraging opportunities [28–31]. In Australia, juvenile male whale sharks aggregate annually at Ningaloo Reef, Western Australia, between March and August [32, 33] coinciding with seasonal blooms of phytoplankton [34]. Bloom activity within the region is believed to be caused by the interaction of warm, southward-flowing tropical waters of the Leeuwin Current, and the cooler, northward-flowing Ningaloo Current [35–37]. These generate an upwelling regime, which creates a dynamic, well-mixed water column that sustains phytoplankton productivity and entrains plankton close to the reef [38, 39]. Whale sharks are known to feed in close proximity to the reef-front at Ningaloo on a variety of prey items including chaetognaths, euphausiids (krill), copepods, stomatopod larvae and small fishes [34, 40–43]. Diet analyses of whale sharks at Ningaloo have identified that euphausiids (krill), namely *Pseudeuphausia latifrons*, form the most significant component of their diet within the region [42, 44] and individuals have been routinely observed feeding on dense swarms of krill in surface waters [41, 45].

The predictability of aggregations at Ningaloo, combined with the frequency of foraging reported in the area provides a unique opportunity to examine the potential mechanistic role of prey density as a driver of whale shark movement patterns. Here, we test whether whale shark horizontal and vertical space use is more concentrated in areas and regions of the water column with higher prey density. We used ship-based acoustic surveys with targeted plankton net tows and CTD (conductivity, temperature and depth) casts to determine the interactions of whale shark movements and the bio-physical environment in which they occurred. These combined measurements enabled us to map the distribution, density and community composition of zooplankton at Ningaloo Reef and provide new insights into the fine-scale (10's m–km) vertical and horizontal movements of tracked whale sharks in relation to bathymetric features and oceanographic processes that may enhance prey density.

## Methods

### Whale shark tracking data

**Fig. 1:**
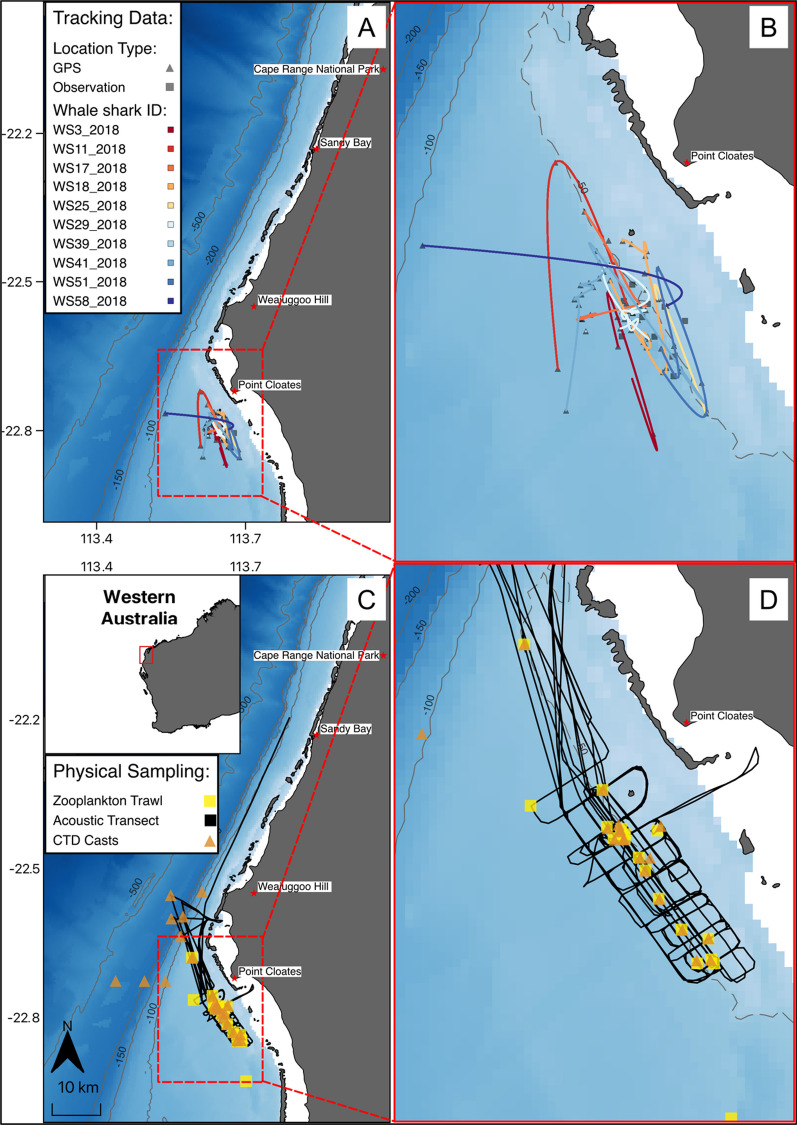
The study area for the satellite tagged whale sharks (**A**) and the area of interest (red rectangle/box) at Point Cloates (**B**), with coloured lines showing the interpolated horizontal movement trajectories of individual whale sharks, squares showing tag deployment locations and triangles showing Fastloc GPS locations. The in-situ physical sampling locations across the study area (**C**), and the area of interest (red rectangle/box) at Point Cloates (**D**) showing the acoustic transects (black line), CTD stations (orange triangles) and zooplankton net trawls (yellow squares). C also shows an overview map of the study area (red rectangle/box) in relation to Western Australia. The dashed grey line indicates the reef edge present at the 50 m bathymetry contour

The study was conducted at Point Cloates (22.7212° S, 113.6775° E) on Ningaloo Reef, Western Australia (Fig. 1A) between the 15th and 24th of May 2018. It involved a coordinated effort between researchers on a 34.9 m research vessel (*RV Solander*) and a team of snorkellers on a 8.4 m charter vessel (Osso Blu). A spotter plane was used to locate whale sharks at the surface and to guide the charter vessel towards the spotted sharks. Snorkelers then entered the water alongside the animal to deploy telemetry tags and collect auxiliary data. Data collection included stereo camera footage to measure shark length, a visual inspection for the presence of claspers to determine sex, and still photos of spot and stripe patterns for individual identification. The tags (SPLASH10-F-323 measuring 225 mm × 71 mm × 76 mm L×W×H from Wildlife Computers, Seattle, Washington) were attached to the dorsal fin via a spring-loaded clamp and a 1.5 m braided stainless-steel trace. The 1.5 m trace allowed the tag antennae to emerge from the water (and so be able to transmit) when the shark swam within approx. 1 m of the surface. A very high frequency (VHF) transmitter was attached to the tag package to aid tag recovery. The clamp was attached (made by Customised Animal Tracking Solutions) by hand to the dorsal fin of the animal, and a galvanic time release was incorporated to detach the clamp and tag from the shark after 24–48 h [46]. Once the clamp detached from the shark, tags floated to the surface and and were tracked and recovered using the latest positions received from the tag and using a hand-held VHF receiver until recovery.

Tags were programmed to sample depth (accuracy ± 1% of measured depth), light intensity (visible band; range of sensitivity 5 × 10^–12^ W cm^−2^ to 5 × 10^–2^ W cm^2^), and temperature (± 0.1 °C) every second, and to obtain location whenever the antenna cleared the surface (as detected by a saltwater switch) for long enough to achieve signal lock with overhead satellites from the Argos satellite system (transmission time is variable depending upon cloud cover, time of day, etc.). In addition to data relayed via Argos satellites, data were also relayed via two mote receiver stations (Wildlife Computers, Seattle, Washington). Motes are autonomous, ground-based receiving stations that increase the number of location estimates when tags are within range (~ 70 km; [47]). These receiver stations were deployed at elevated sites at Sandy Bay (5 m above sea level), Cape Range National Park and Weajuggoo Hill (143 m above sea level) on Ningaloo Station (Fig. 1). The additional locations provided by the motes assisted with tag recovery.

After floating tags were recovered, the tag was downloaded and the data uploaded to the Wildlife Computers Portal for processing of the dive/temperature data and GPS locations. The resulting tracking dataset was filtered to include only location estimates when the tag was attached to the shark. Tags were assumed to be detached and floating when the acquisition of locations matched the time intervals (every 30 min) programmed to obtain a location fix. From the filtered dataset, we then calculated the time each shark spent at every 1 m of depth (TAD; time at depth) and every 1 °C of temperature (TAT; time at temperature) to quantify shark depth use and examine patterns of temperature use.

### Prey distribution and oceanography data

Acoustic transect surveys were conducted using a Simrad EK60 General Purpose Transceiver (GPT) with a 120 kHz split beam transducer (ES120-7G 7°beam width) onboard the RV Solander. The echosounder transducer was mounted using a custom-made bracket on the internal moonpool (600 × 600 mm), which positioned the transducer on the starboard side of the vessel, 2.7 m below the water surface. All acoustic data were collected during the daytime, at a nominal vessel speed of 10 knots and logged through Simrad ER60 software (v2.4.3). The echosounder pulse duration was set at 1024 μs (power 500 W) with the ping interval of 0.5 s and a 200 m maximum recording range. The sounder was calibrated using a tungsten carbide sphere (38.1 mm in diameter) following the standard procedure outlined by Demer et al*.* [48] and temperature (27.5 °C) and salinity (35.2) data recorded from a CTD cast at the calibration site.

Daytime echosounder surveys consisted of a series of parallel transects (~ 3.5 km lines, line spacing ~ 1 km) running perpendicular to the reef edge to capture the variability in bathymetry (complex topography from 10 to approximately 50 m depths and adjacent low relief seabed > 50 m depths) and maximise sampling effort in locations where whale sharks were commonly encountered. Intermittently (generally towards the start and end of each day or when a shark was encountered), transects were also run parallel to the reef to document prey densities along the complex bathymetry or to intersect the last known position of a shark (Fig. 1B). When locations of whale sharks were communicated via the spotter plane or deployed tag, transects were conducted around the position in a box-like grid (~ 500 m lines) to map the prey field in the vicinity of the shark. An average of 13 echosounder transects were conducted in an area covering ~ 60 km^2^. Transect lines ranged in distance from 97 to 141 km per day covering an average depth of 45 m (Fig. 1B).

We used a 30 cm diameter bongo net (mesh size of 355 μm) to trawl/sample targets deemed to be krill based on their acoustic signature in the echosounder. Trawl nets were deployed (19 in total from 18/05/2018 to 24/05/2018) each time a discrete, intense scattering signature of dense patches of krill were detected. The position of the discrete acoustic scattering was marked, and the ship returned to this point in the transect line for a target trawl. During net trawls the vessel was slowed, and the corresponding acoustic data was removed from the analysis examining the relationship between sharks and prey. The bongo net was towed for 10–25 min either obliquely through the entire water column or horizontally at sampling depths selected according to the mean depth of the target in the echosounder. The net haul speed did not exceed 20 m per minute and the volume of water filtered ranged between 10 and 200 m^3^ depending on the depth of the target. To ensure all samples were concentrated in the cod end of the net, nets were rinsed down with seawater when retrieved. Zooplankton were then transferred to containers for preservation in formaldehyde or in ethanol, or frozen in liquid nitrogen. Preserved samples were analysed for community composition (using Zooscan to determine the major taxonomic classes), abundance and biomass using microscopy and a laser optical plankton counter (LOPC; Rolls-Royce, England). A total of 18 net tows were sampled at depths ranging from 5 to 45 m.

All net tows were followed by CTD casts using a Livewire CTD carousel equipped with a SBE911plus CTD that sampled temperature (°C) and conductivity (S/m) at 24 Hz throughout the water column. Salinity was calculated from measurements of temperature, conductivity and pressure. Chlorophyll-a (μg L^−1^) was measured using an Wetlabs Eco Triplet operating at 4 Hz on board the ship.

Bathymetry data were obtained from the general bathymetric charts of the oceans (GEBCO 2018), at a resolution of 30 arc-second interval grids. High-resolution (3-m) bathymetry was sourced from Geoscience Australia for the Black Rock and Point Cloates area. The area along the 50 m bathymetry contour was defined as the reef edge (Additional file 4: Fig. S1).

### Echosounder data analysis

Following seabed detection and removal of near-surface bubble entrainment (data cleaning), single-ping backscatter data (1 m vertical bin size) was processed in MATLAB and echo integrated using along-track intervals of 30 s (equivalent to c. 150 m of transect line) and 1 m bins vertically (MVBS, hereafter S_v_; dB re 1 m^−1^; logarithmic, see Maclennan et al*.* [49] for definitions of acoustic variables) from ~ 5 m depth to the seabed. We calculated the nautical area scattering coefficient (NASC; m^2^ nm^−2^), a measure of the cumulative backscattering through a layer of water, using 500 m intervals along the transect, as described by Maclennan et al*.* [49]. The echo integrated S_v_ data was scaled to krill density (as krill are a significant portion of whale shark diet in the region; [42]) using a target strength (TS, in dB re 1 m^2^) of − 88.53 dB at 120 kHz; this was calculated using the observed length-distribution of 100 krill individuals (size range 8–13 mm) and applying the distorted wave Born approximation (DWBA) scattering model using the Zooscat R package [50]. From this, the volumetric density (individuals per m^3^) was calculated per depth bin of 1 m.

### Calculating whale shark habitat use 

To quantify associations between habitat use by whale sharks and prey distribution and density, we first estimated the general horizontal area used by the sharks by calculating a utilisation distribution (UD; 50% KUD ~ core and 95% UD ~ range area) using the R package ‘adehabitatHR’ [51] for all sharks combined with location points from all GPS positions of the shark at tag deployment and locations from the satellite tags (deployment and GPS locations from the satellite tags). Estimation of utilisation distributions is a common approach to measuring the space use of animals. It provides a probability density that an individual can be found at a certain point in space [52]. We then used the UD values per grid cell (grid size = 500 × 500 m) for data pooled for all sharks as the response variable in a spatial modelling framework (outlined below) with NASC per grid cell (dB re 1(m^2^ nmi^2^); NASC) as the predictor variable.

To incorporate vertical movements into the utilisation distribution estimate, we used the R package ‘KUD3D’ (https://github.com/vinayudyawer/KUD3D) after time matching the position estimates from the tags with the high-resolution temperature and depth record. We used a spline interpolation between successive location estimates (GPS location only) to generate an estimated spatial position for each data point in the temperature and depth records (recorded every second). Although movement models exist to provide more realistic movement paths of animals (e.g., dead reckoning), our tag deployments were of too short duration (usually < 24 h) and had too few position estimates per individual to obtain model convergence.

### Whale shark movement and prey

To examine the 3D movements of whale sharks in relation to their prey, we first matched data sets of spatial and temporal movements of sharks with the data obtained from the acoustic surveys. Initially, we filtered the shark tracking dataset to derive a subset that matched the timing of the acoustic surveys. We then spatially matched these tracking and acoustic datasets using the ‘sp’ R package by creating a 1 km spatial buffer around each shark location point and extracting the points from the acoustic survey within that buffer zone. We chose a 1 km spatial buffer as we identified spatial autocorrelation in acoustic backscattering intensity up to 1 km between points in the acoustic survey by fitting a variogram model with a gaussian distribution using the ‘gstat’ R package (Additional file 4: Fig. S2; [53]). Acoustic estimates of prey density (individuals per ${{\text{m}}}^{3}$) were then averaged to calculate the mean density (individuals per ${{\text{m}}}^{3}$) of the prey field within the buffer zone for each location point for the shark throughout the water column using 1 m depth bins and included depth layers that contained prey density (individuals per ${{\text{m}}}^{3}$) measurements of 0.

To examine whether depth use by tagged sharks was driven by prey density, we developed generalised linear mixed effects models (GLMMs) using the ‘*lme4*’ R package [54] for the full data set (5 m to the seabed) and for data within the depth ranges of 5 m to 50 m to investigate differences between shallow (< 50 m) and deep water habitats (> 50 m). We included shark TAD as the response, averaged prey density (individuals per m^3^) as the predictor variable, and shark ID as a random effect to avoid pseudo-replication and to enable relationships to vary among individuals. We used the Akaike’s Information Criterion weight (wAIC; [55]) to compare our model with the null model (TAD ~ 1 + shark ID). Model assumptions and fits were checked using diagnostic plots following Zuur et al*.* [56].

In the results section below, all values reported in brackets are means ± standard deviations (SD) unless otherwise stated. 2D utilisation distributions are reported as areas whereas 3D utilisation distributions are reported as volumes.

## Results

### Whale shark tracking

We identified a total of 40 individual sharks across 79 sightings (some sharks were re-sighted), with total shark length ranging from 3.00 to 9.00 m (mean 5.61 ± 1.30 m; Additional file 4: Table S1). Of these individuals, 68% were males, 17% were females and 15% were of unknown sex. Based on the presence of calcified claspers [57], only nine males (1.2%) were mature. We successfully deployed tags on 12 juvenile sharks consisting of 11 males and one female ranging from 5 to 8 m TL (mean ~ 6.45 ± 1.12 m; Table 1). Of the 12 tags deployed, two detached prematurely (< 1 h) and data from these deployments were not included in the analyses. Deployment durations ranged from 13.71 to 36.28 h (mean 22.42 ± 7.8 h) and provided a total of 234 h of depth, temperature and light data recorded at 1 s intervals. Location estimates obtained per deployment ranged from 2 to 17 (7.66 ± 5.02 locations; Table 1). The mean straight-line distance travelled by tagged sharks from the start to the end of deployment was 2.88 ± 2.63 km, ranging from 0.30 to 13.66 km. All tagged sharks remained within the general vicinity of Point Cloates (Fig. 1A).

**Table 1:** Summary statistics of the tracking dataset for each tagged whale shark. M = Male, F= Female, AWST = Australian Western Standard Time

Whale shark ID	Sex	Size (m)	No. of locations	Tracking duration (hours)	Start date (AWST)	End date (AWST)
WS3_2018	M	6	4	13.8	15/5/2018 13:17:02	16/5/2018 3:03:00
WS11_2018	M	8	4	14.6	16/5/2018 13:20:02	17/5/2018 3:55:00
WS17_2018	M	7	8	14.9	17/5/2018 14:16:00	18/5/2018 5:12:00
WS18_2018	F	7	9	24.3	18/5/2018 12:51:00	19/5/2018 13:10:27
WS25_2018	M	6	4	22.6	19/5/2018 11:20:00	20/5/2018 9:58:01
WS29_2018	M	6	17	26.9	19/5/2018 11:55:00	20/5/2018 14:46:00
WS39_2018	M	NA	2	36.3	20/5/2018 11:43:00	21/5/2018 23:59:59
WS41_2018	M	6	14	20.9	20/5/2018 13:04:00	21/5/2018 9:55:15
WS51_2018	M	8	7	34.5	21/5/2018 13:27:01	22/5/2018 23:59:59
WS58_2018	M	6	4	25.5	22/5/2018 12:43:00	23/5/2018 14:12:58
Total (mean):		6.6	7.3	22.4		

**Fig. 2:**
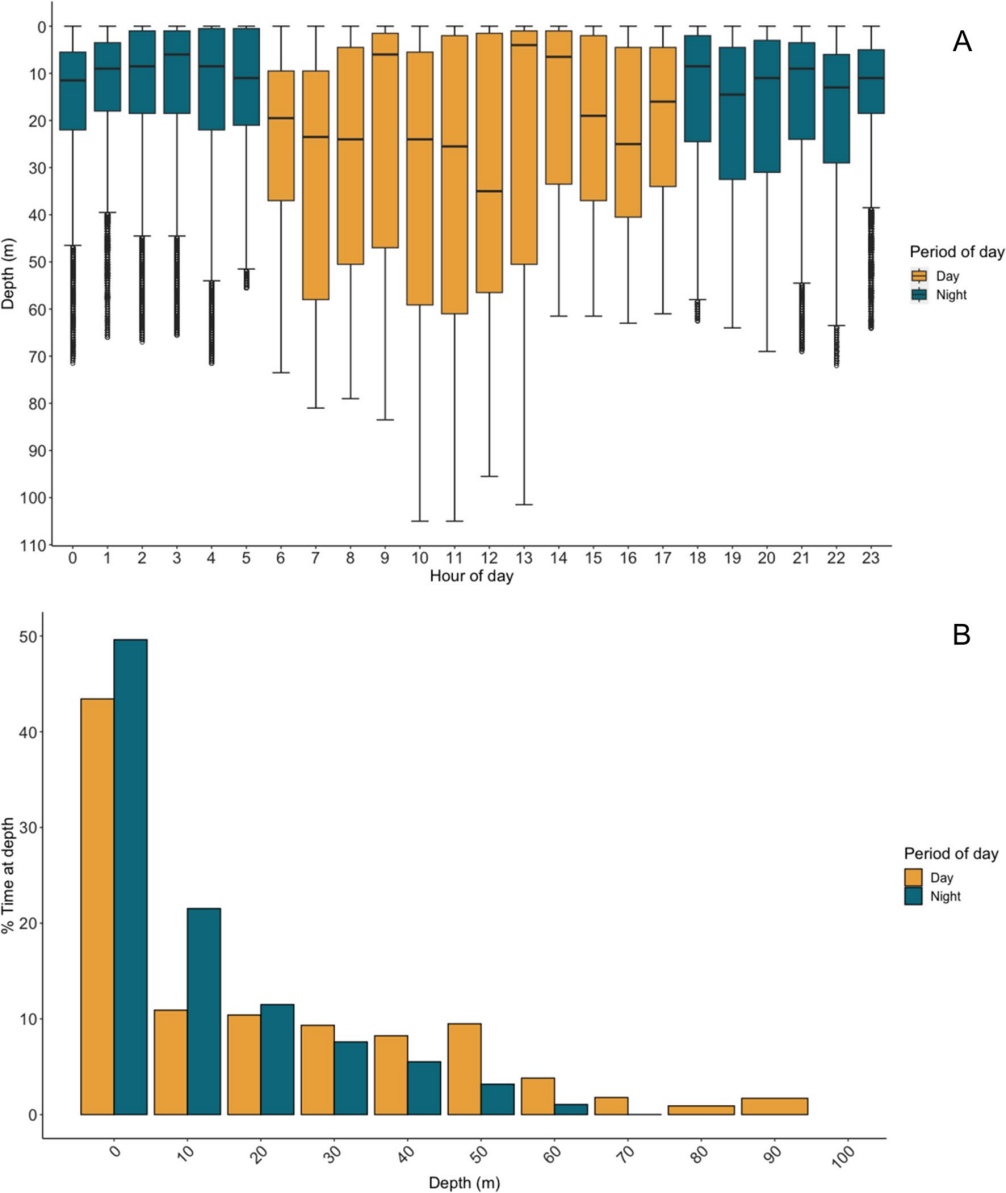
**A** Depth use of all tagged whale sharks (day and night) calculated from the 1s sampled depth data from the tags. Boxplots depict the lower quartile, upper quartile (and thus the interquartile range), and median within the data, with whiskers extending from the shallowest to the deepest depths recorded. **B** Histogram of % of time sharks spent per 10 m depth bin throughout the water column (day and night)

Whale sharks used a greater range of depths during the day (mean = 25.46 m, median = 18.00 m, range = 0–105 m) than at night (mean = 15.31 m, median = 10.50 m, range = 0–72 m; Fig. 2A). Sharks spent ~ 50% of time in the upper 10 m of the water column during both day and night (Fig. 2), with a second peak at depths of 50–60 m during the day (Fig. 2B). The time series (1 s) of temperature showed that sharks experienced a mean water temperature of 27.12 °C (± 0.30 ºC). During day light hours, sharks spent 100% of time within the temperature bin of 28 °C (Additional file 4: Fig. S3), over a depth range from the surface (< 5 m) to > 100 m (Fig. 2A). During night hours, the TAT data showed that sharks spent 20% of time at 27 °C and 80% of time at 28 °C (Additional file 4: Fig. S3), over a depth range from the surface (< 5 m) to > 70 m (Fig. 2A).

### Prey distribution and oceanographic conditions

**Fig. 3:**
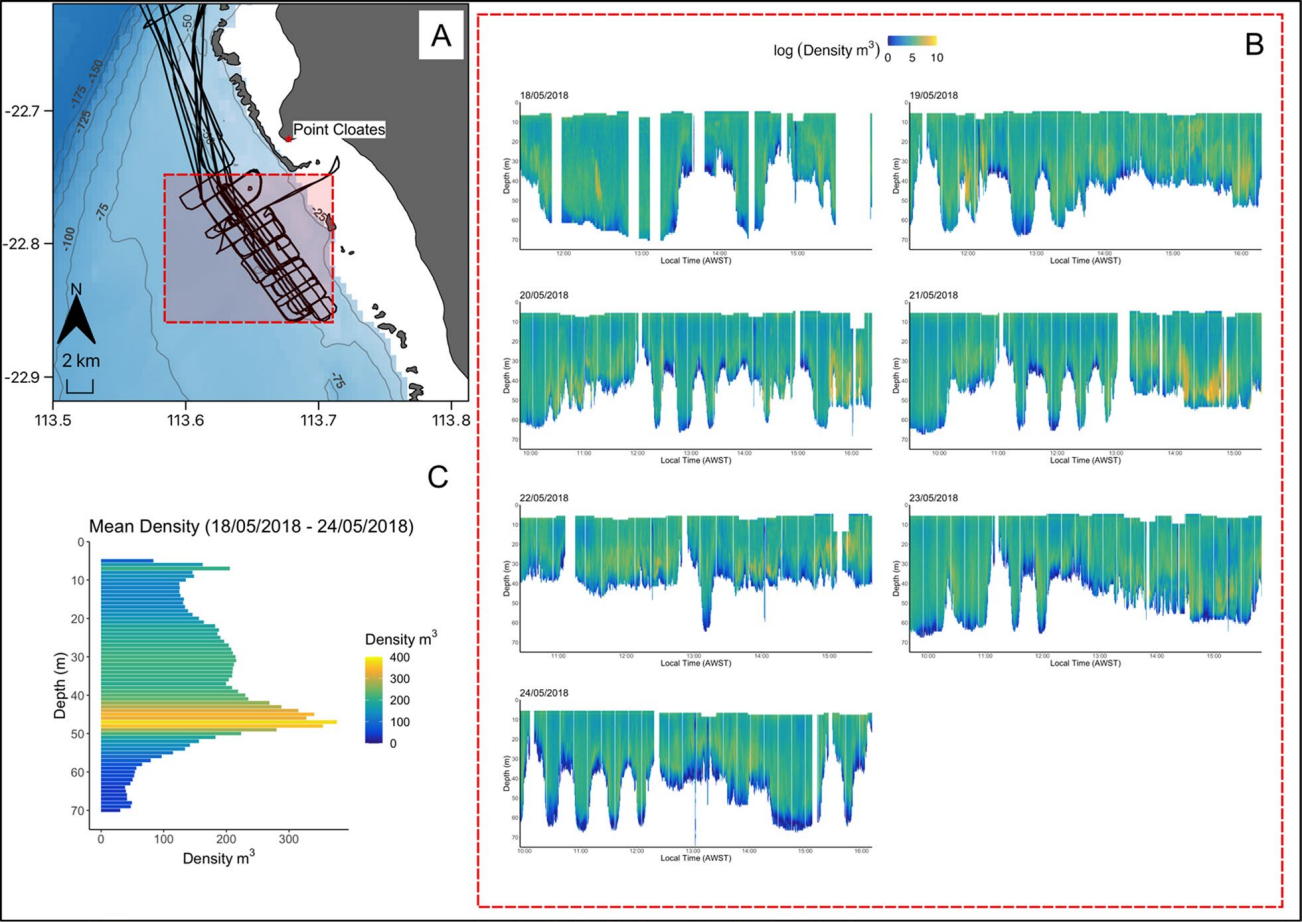
**A** Map showing the acoustic transect lines with the area of focus shaded in red and the grey lines indicating the bathymetry contour per 25 m increments. **B** The 2D log prey density (individuals per m^3^) in the water column from 5 m to the seabed from 18/05/2018 to 24/05/2018 within the focus area. (**C**) A histogram of average prey density (individuals per m^3^) from 19/05/2018 to 24/05/2018 per 1 m of depth throughout the water column within the focus area. No data were available for the top 5 m of the water column (see methods for transducer depth). Logarithms were calculated with base_e_

Prey biomass derived from targeted plankton tows ranged from 0.12 to 6.09 mg/${{\text{m}}}^{-3}$ (1.77 ± 1.56) with a mean abundance of 2.23 ± 0.95 individuals/L. Backscatter intensities revealed zooplankton-like scattering from 5 to 288 m depths (45.50 ± 44.16). Prey densities estimated from acoustic surveys were variable both horizontally and vertically and over time, ranging from 0.01 to 238,666.75 $\mathrm{individuals~per }~{{\text{m}}}^{3}$. High densities of prey (207.19 ± 724.01 $\mathrm{individuals~per }~{{\text{m}}}^{3}$) were identified across a range of depths throughout the water column, however, were often associated with bathymetric features such as small pinnacles, ridgelines, gutters and complex bathymetry within the 40–50 m depth contours and areas adjacent to reef passages (Fig. 3B & C). Net tows identified three main taxa of zooplankton, which were calanoid copepods (56.8%) tropical krill (*Pseudeuphausia latifrons*; 27.4%) and cyclopoid copepods (7.6%).

**Fig. 4:**
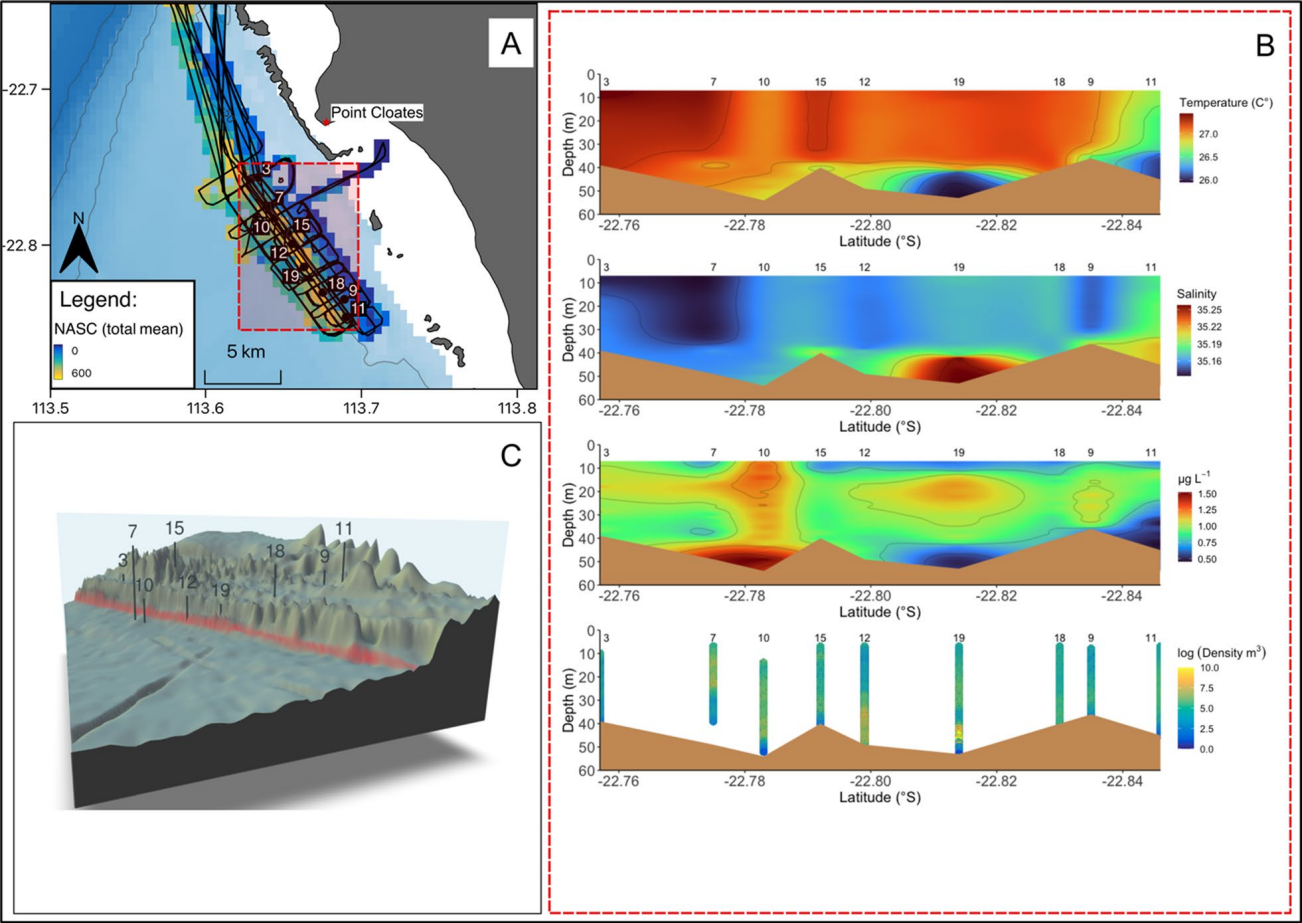
**A** Map showing the acoustic transect lines and area of focus in the red square plotted over the gridded NASC (m^2^ nm^−2^) data. The numbers represent the CTD casts of interest and corresponds to panel C of this figure. CTD casts were conducted from 18/05/2018 to 24/05/2018. **B** interpolated CTD-derived data plotted along the latitudinal sampling gradient with bathymetry derived from GEBCO (brown shaded areas) and CTD cast number (above each plot) shown for each of the four plots (starting at the top) displaying temperature (°C), salinity, chlorophyll-a (µg L^−1^) and the corresponding vertical profiles of acoustic measurements of log prey density (individuals per m^3^) within the area of interest. **C** CTD transect station locations within the area of interest plotted over 3D bathymetry. The red line represents the reef edge situated along the 50 m contour line of the bathymetry.Logarithms were calculated with base_e_

CTD casts were made along the reef with bottom depths ranging from 7 to 60 m (mean ~ 35.85 m; Figs. 4A & B). CTD profiles revealed a well-mixed water column across the shelf with temperatures varying from 26.0 to 28.0 °C and uniform salinity (range from 35.10 to 35.25) from the surface to > 60 m depths (Fig. 4B). Between the 25 and 50 m depth contours and in areas adjacent to the large reef passage (CTD 7,9,10,11,12,18 and 19; Fig. 4C) which separates the reef slope from the lagoon, mixing was evident in the top 30 m with regions of vertical stratification (in both temperature and salinity) associated with deeper parts of the complex bathymetry (reef gutters and pinnacles at 40–50 m depth; Fig. 4C). Here, warmer (27.5 °C), lower salinity (35.15) surface waters overlaid cooler (26 °C) and more saline (35.25) water near the seabed (Fig. 4B). This cooler, higher salinity water extended to ~ 20 m above the seabed near the reef edge in some places. Values of chlorophyll-a ranged from 0.4 to 1.5 µg L^−1^ along the reef edge (Fig. 4B) and generally formed a subsurface maximum just above the seabed in water depths between 10 and 50 m. Values of chlorophyll-a above 1 µg L^−1^ where closely associated with the colder and more saline bottom waters (at around latitude 22.81, see Fig. 4B) and attained a maximum of 1.5 µg L^−1^ (near latitude 22.78, Fig. 4B). These maximum values were associated with a frontal system of warmer (27.5 °C), lower salinity (35.15) surface waters.

### Habitat use by whale sharks

**Fig. 5:**
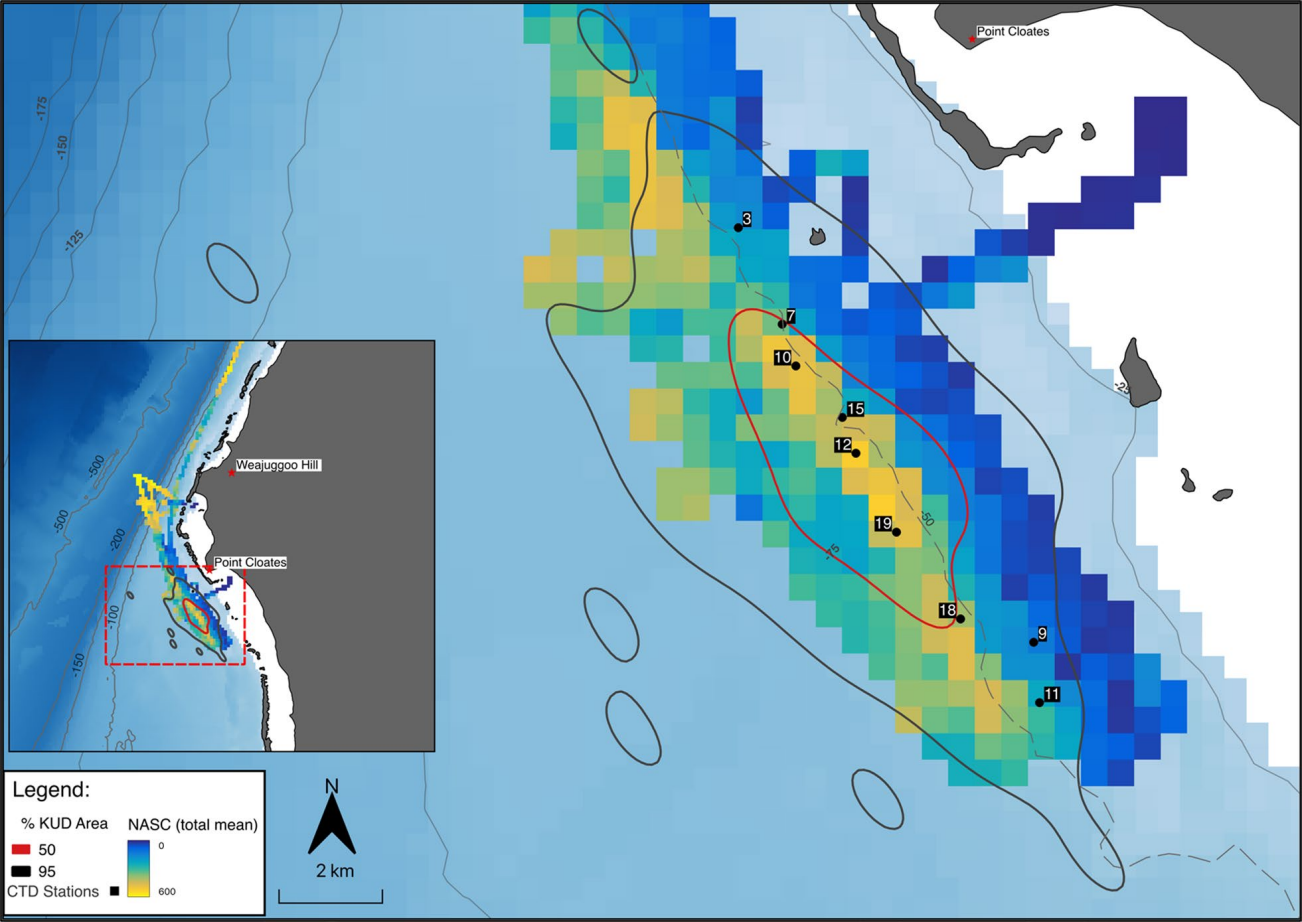
Kernel utilisation density (UD) of tracked whale sharks (50% UD in the red line and 95% UD in the black line) in relation to gridded NASC (m^2^ nm−^2^), bathymetry (contours shown in thin, grey lines) and the reef edge at 50 m (dashed grey line) along the Ningaloo Reef. Also shown is the CTD station numbers of interest. Insert map shows whale shark 50% and 95% UD contours in relation to NASC (m^2^ nm^−2^) over a larger extent showing the area in focus within the dashed red square

A total of 74 position estimates (64 GPS locations from satellite transmitters and 10 from GPS positions taken at tag deployment using handheld GPS) were used to calculate 2D utilisation distributions. The extent of the area used by whale sharks (95% 2D UD) spanned 274 km^2^ and was centred in the offshore waters around or south of Point Cloates (Fig. 5). The core area (50% 2D UD) of use was concentrated in a small area (22 km^2^) parallel to the reef edge and slightly south of Point Cloates, adjacent to a large passage in the reef.

**Fig. 6:**
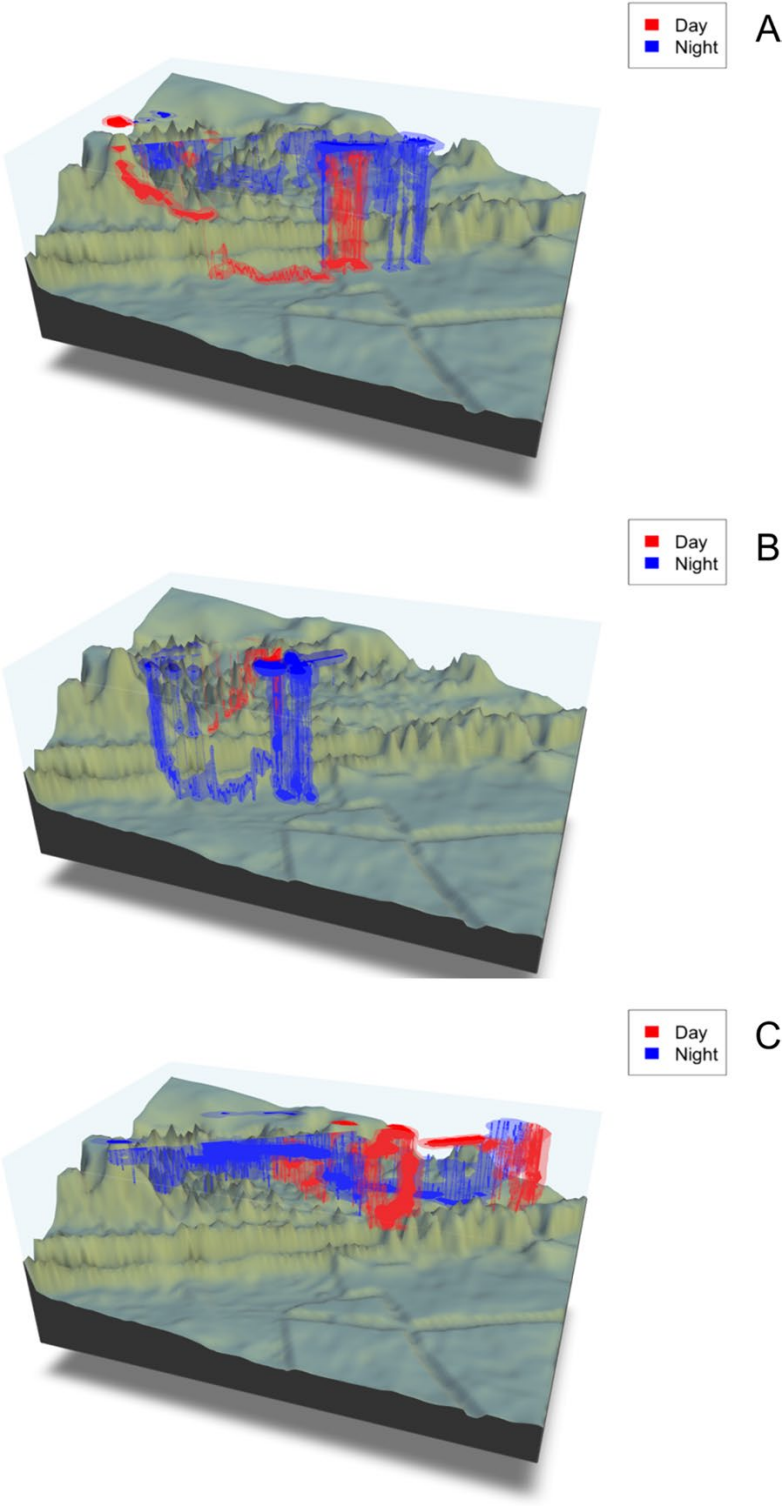
The 50% (darker shades) and 95% (lighter shades) three-dimensional UD of tracked whale sharks; WS18_2018 (**A**), WS29_2018 (**B**) and WS51_2018 (**C**) split by day (red) and night (blue) in relation to the bathymetry. For interactive html 3D models, please see Additional files 1 for WS18_2018 (**A**), 2 for WS29_2018 (**B**) and 3 for WS51_2018 (**C**)

The 3D utilisation distribution of each tagged shark encompassed the entire water column from surface to the seafloor (Fig. 6 & Additional file 4: Fig. S4). The core space (50% 3D UD) use covered an average of 0.07 ± 0.04 km^3^ and comprised a relatively small portion of the 3D volume occupied by tagged individuals (95% 3D UD), which covered 0.57 ± 0.32 km^3^ (Fig. 6 & Additional file 4: Fig. S4). The mean 3D core (50% 3D UD) and range (95% 3D UD) of space use by sharks did not vary significantly between day and night (Wilcoxon rank sum test: W = 1078, p = 0.38). All sharks demonstrated extensive use of the surface zone, with the core space use (50% 3D UD) within the upper 10 m of the water column (Fig. 6 & Additional file 4: Fig. S4) during both day and night portions of their tracks. Additionally, all sharks displayed core space (50% 3D UD) use in 40–60 m depths parallel to the reef edge where there were gutters, small pinnacles and ridgelines in the bathymetry (Fig. 6; Additional file 4: Fig. S4).

### Whale shark movement and prey

**Fig. 7:**
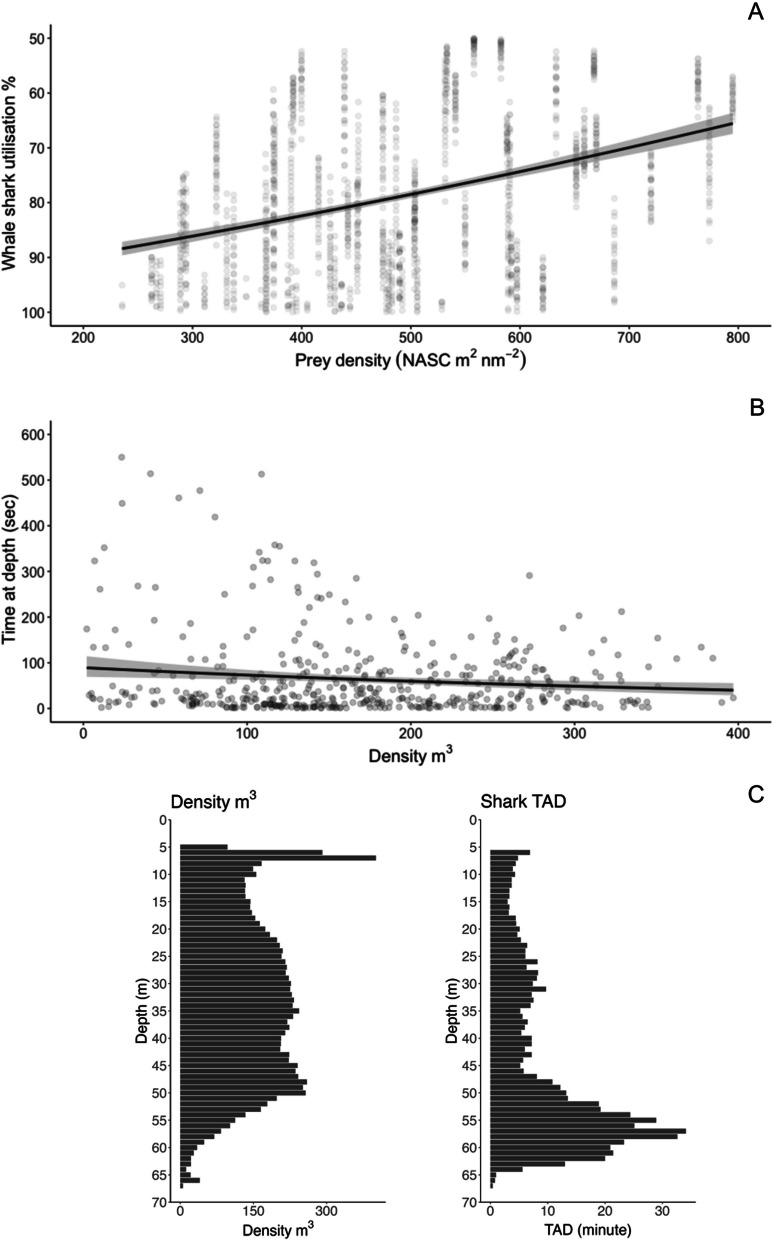
**A** Fitted line from the GLMM with NASC as the predictor and shark utilisation distribution (core ~ 50% UD, entire extent ~ 95% UD) as the response with the data points and 95% confidence interval (grey shading around the fitted line). **B** Fitted line for the GLMM model for the entire water column with prey density (individuals per m^3^) as the predictor and shark TAD per 1 m depth bins as the response with the data points and 95% confidence interval (grey shading around the fitted line). **C** Histograms of acoustically derived mean prey density (individuals per m^3^) measured within the 1 km buffer of each shark spatial point and shark TAD (minute) for periods of the track spatially and temporally match with the acoustic transect

Prey biomass was higher within the core area (50% 2D UD) with a median of 0.76 g ${{\text{m}}}^{-3}$ (range 0.18–3.81 g ${{\text{m}}}^{-3}$) compared to the range (95% UD), which had a median of 0.57 g ${{\text{m}}}^{-3}$ (range 0.13–2.22 g ${{\text{m}}}^{-3}$; Additional file 4: Fig. S4). Similarly, prey abundance (individuals L^−1^) was also higher in the core area (50% 2D UD), which contained a median of 1.54 individuals L^−1^ (range 0.45–4.58 individuals L^−1^) compared to the area of the use range (95% 2D UD) where there was a median of 0.84 individuals L^−1^ (range 01.8–2.78 individuals L^−1^; Additional file 4: Fig. S4). The GLMM model revealed a positive relationship (r^2^ = 0.15; Fig. 7A) between prey densities (as expressed by NASC) and the core area (50% UD) use by whale sharks.

Movements of 6 individual sharks could be spatially and temporally matched with the acoustic transects. There was no relationship between vertical movements of sharks (TAD) and prey densities throughout the entire the water column (r^2^ = 0.02; Fig. 7B) or within shallow water environments (r^2^ = 0.02; Additional file 4: Fig. S6). However, smaller peaks of whale shark TAD around 25–30 m and 45–60 m depths matched peaks in prey density (Fig. 7C). In addition, the random effect (shark ID) in our model accounted for 35% of variance in the relationship between shark TAD and prey density.

## Discussion

Our spatial modelling found that the horizontal (2D) utilisation distributions of whale sharks were strongly associated with areas of elevated prey densities, as measured by both net trawls and acoustic transects. Although whale sharks dove to depths containing high prey density, our modelling found that whale sharks did not extend their time at depths where prey densities were elevated. Horizontal areas of high use by sharks were concentrated in waters off the reef edge between 50 and 60 m depths and to the south of Point Cloates adjacent to a large reef passage that separates the reef slope from the lagoon. CTD casts revealed that the vertical structure of the water column in these areas was characterised by bands of colder, high salinity water near the seabed most likely exiting the lagoon at depth as dense water transport [58]. The upper water column in this area contained higher values of chlorophyll-a and increased prey densities approximately 20 m above the seabed, suggesting that bathymetric features combined with these deep-water flows aggregated and retained prey for sharks at depth. Therefore, our combined in situ measurements add some support to previous studies that have suggested that horizontal patterns of movement of whale sharks are associated with bio-physical processes that enhance productivity and concentrate prey biomass [59–64].

Other studies have also found a close association between horizontal (2D) movement patterns of whale sharks and their prey at scales of 1–10 s of kilometres, particularly where whale sharks aggregate to feed on prey originating from a limited source area such as spawning schools of fishes [28, 65] or krill swarms [29, 66]. The core area of use by whale sharks at Ningaloo Reef was directly adjacent to a large reef passage slightly south of Point Cloates and is a predictable “hotspot” of whale shark abundance, with high numbers recorded there over many years [67, 68]. The presence of these dense water pockets also supports larger-scale studies of reef passages at Ningaloo, where oceanographic frontal zones due to physical forcing mechanisms (e.g., tides, waves and wind) flush more dense lagoon water towards the shelf where it mixes with shelf water and enhances productivity [45, 69]. In addition, a mesoscale eddy system also occurs in this area in waters off the reef [35, 70] that is likely to both physically aggregate prey and create upwelling conditions suitable to enhance primary production in the upper water column. Similar to other epipelagic marine predators [71–73], whale sharks utilise mesoscale eddy currents to locate and consume prey resources [74, 75]. Together, our results and those of earlier studies suggests that the core use area of whale sharks south of Point Cloates is likely a result of physical forcing, which creates an intense mixing regime along the reef edge that promotes productivity, thereby concentrating prey biomass at both meso- and local scales.

**Fig. 8:**
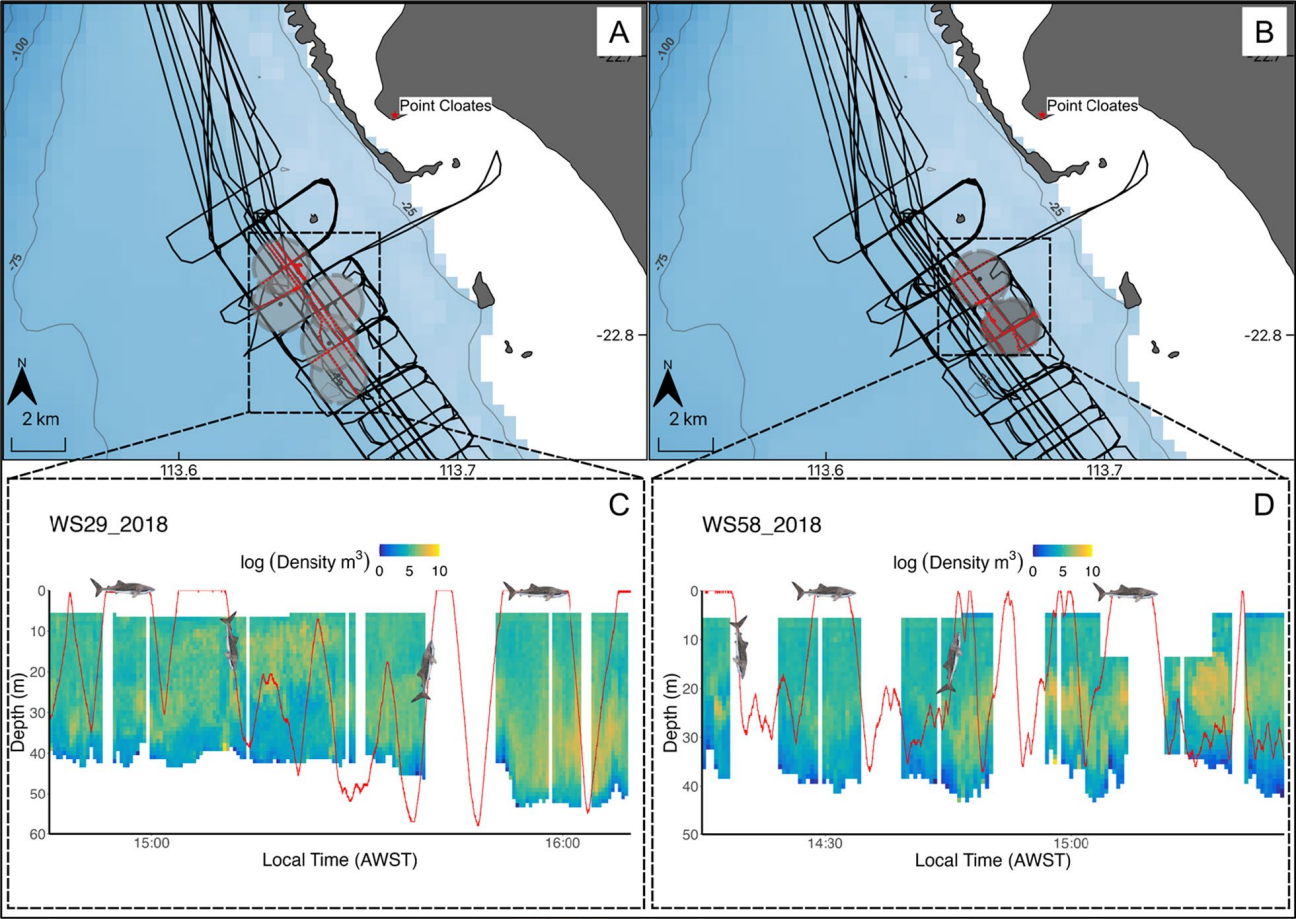
Map showing areas of spatially and temporally matched acoustic transects (red dots overlaid on black lines) which fell within a 1 km buffer (grey circles) of each shark’s location, for shark WS29_2018 (**A**) and WS58_2018 (**B**), respectively. Corresponding insets in C and D show a time series plot of extracted log prey density (individuals per m^3^) from within the 1 km buffer of each shark’s spatial point and temporally matched with the dive profile (red line) of shark WS29_2018 (**C**) and WS58_2018 (**D**). Logarithms were calculated with base_e_

In the vertical dimension, relationships between whale sharks and prey densities were more complex. Prey was distributed throughout the water column, but was most abundant at approximately 40–50 m depths, broadly matching water column depths targeted by whale sharks in their repeated patterns of descents from the surface (Fig. 8). Despite these observations, our results found no quantitative relationship between density measurements of prey (which excluded the near-surface < 5 m depths) and whale shark TAD, except for sharks spending more time in deeper water during the day than at night (Fig. 2). To some extent this diel pattern of depth use aligns with the diel vertical migration patterns of zooplankton prey (e.g., shallow at night, deeper during the day; [76]) both in coastal and open ocean environments [77–82]. It is important to note, however, that we were unable to quantify prey density in the upper 5 m of the water column due to transducer depth (5 m depth; see methods), a problem inherent to echosounders fixed to large vessels. This limited our ability to investigate the potential relationships between whale sharks and their prey at the surface. Given that krill form dense swarms at the surface at Ningaloo [83] where whale sharks are often observed feeding [41, 84], the quantification of zooplankton at the sea surface would be important to understand drivers of surface use. However, it is not possible to sample this layer using downward-facing echosounders mounted on the ship due to the blanking distance and entrainment of air bubbles near the surface, which increases with wind and wave action [85]. Even if other methods were available to quantify prey density at the surface (e.g. net trawls), the challenges of conducting ship board operations at night (when zooplankton typically rise to the surface), near the reef and in the vicinity of surface feeding animals [41] would make such sampling extremely difficult.

Within areas of elevated productivity, marine predators are known to adapt foraging strategies to track and target depths with high prey concentrations [23, 86, 87]. For example, blue whales (*Balaenoptera musculus*) reduce foraging frequency when prey densities are low to minimize oxygen use [22] and target depths that consist of the highest density prey patches [15]. Likewise, basking sharks (*Cetorhinus maximus*) reduce swim speeds [88] and modify dive behaviours to feed on prey above a certain density threshold [24, 89] . Our choice of response variable (TAD) assumed that similar behaviours existed in whale sharks, however, we found no evidence to support the notion that whale sharks extend their time spent at specific depths away from the surface to increase prey consumption at Ningaloo Reef. Although shark TAD did not increase with prey density, this discrepancy may be explained by a more limited extent of prey patches at finer spatial scales (< 1 km) or by a temporal mismatch between the measurement of mobile prey and shark utilisation. Nonetheless, individuals tended to make repeated ascents and descents through the water column, sometimes coinciding with high density patches of prey but did not spend more time at depths (TAD) where prey density was highest. This finding was consistent with Cade et al*.* [26] who showed that when foraging at depth, whale sharks maintained a fixed, slow swimming speed, rather than rapid accelerations and posture changes which would be more indicative of targeted feeding events or the efficient capture of mobile prey. It could be that active ram or gulp filter feeding at depth may be less successful or more energetically costly for this species (e.g. due to krill avoidance behaviour). Unlike the great whales, which can adapt to dynamic changes in prey field structure through rapid changes in body orientation and swim speeds [90, 91], whale sharks are constrained by their body plan [82] and must maintain a slow swim speed and incorporate glides into their descents to maintain foraging efficiency [26, 82, 84]. Perhaps the energetic constraints imposed by this body plan limit their ability to remain in particular depths tracking prey and thus limit their effectiveness of harvesting mobile, fast-swimming prey, such as krill [92, 93], that can actively avoid predation by such slow moving predators [94]. In contrast, prey that is effectively trapped at the water–air boundary may be more accessible and more easily captured using ram filtration or gulping. If this is the case, whale shark vertical movements may be driven simply by the prey being available regardless of prey density given that this species is a slow swimming and somewhat indiscriminate ram filter feeder. This could explain why whale sharks maintain a pattern of ascents and descents through the water column irrespective of the presence of prey at depth (Fig. 8), even though as gill breathers, there is no requirement to return to the surface, unlike lung-breathing marine megafauna.

Prey densities at Ningaloo Reef were similar to those recorded at other aggregation sites [29, 61, 79, 95] and were within the broad range at which whale sharks have been observed foraging [96–98]. Net tows revealed calanoid copepods (56.8% of total catch) as the most prominent zooplankton taxa followed by tropical krill (27.4% of total catch) and cyclopoid copepods (7.6%). Notably, the proportion of krill in the total catch was significantly lower than the estimates provided by Wilson et al*.* [99], who reported krill (*Pseudeuphausia latifrons*) as the predominant microzooplankton species at Ningaloo Reef when sampled using light traps [100]. This disparity in krill abundance is likely a result of the differences in sampling protocols, as net avoidance behaviours by krill can lead to significantly lower estimates of abundance and biomass compared to alternative methods [101, 102]. Nonetheless, consistent with earlier studies [45], acoustic surveys showed that densities of prey patches were highly variable (range from 0.01 to > 200,000 $\mathrm{individuals~per }~{{\text{m}}}^{3}$) over short spatial (meters) and temporal (seconds) scales, but were generally denser at depths of 40–60 m, corresponding to bathymetric features. Bouchet et al*.* [103] suggested that areas with complex bathymetric features, such as small pinnacles, seamounts, channel structures and ridgelines accumulate or retain zooplankton that could subsequently attract filter feeders, particularly at epipelagic depths. Our analysis of 3D utilisation distributions supported this hypothesis, as it illustrated the tendency of sharks to target these features along the seabed. However, only a relatively small portion of the 3D core space use (50% 3D utilisation distribution) occurred at depth, with the majority occupying near-surface (< 5 m) waters (Fig. 6). As noted previously, this may reflect differences in foraging efficiency of whale sharks across the water column.

While diving, whale sharks repeatedly traversed dense aggregations of prey (Fig. 8C & D), presumably ram filter feeding on both descents and ascents. A strategy of largely indiscriminate filter feeding during dives is consistent with the observation that sharks may ingest macroalgae such as *Sargassum* while foraging [104]. If our suggestion that greater feeding efficiency drives the frequent return of these sharks to the surface, it implies that feeding at the surface offers an energy return even greater than foraging during a gliding descent, when the energy output of active swimming is not required. The suggestion that whale sharks forage at the surface is consistent with observations of the predictable onset of very active ram filter feeding by whale sharks at dusk, when vertically migrating zooplankton move to surface waters in coral reef environments [84, 105]. Gleiss et al*.* [84] suggested that surface ram filter feeding at this time could provide a significant part of the energy requirements of these animals.

## Conclusion

Our study provides the first (to our knowledge) on the sub-surface movements of whale sharks in relation to their zooplankton prey. We found a positive relationship between whale shark horizontal space use and prey density, supporting the hypothesis that whale sharks aggregate along areas of Ningaloo Reef that support higher concentrations of prey. These areas were associated with a combination of complex seafloor topography (pinnacles and reef gutters) and localised current flows near reef passes. However, we found no quantitative evidence to suggest that sharks target, and extend their time spent at depths across the water column that contain the highest densities of prey irrespective of the observation that sharks repeatedly dove to these depth layers. Future studies that can map prey fields throughout the entire water column, including near the surface such as optical [106] and sonar tags [107] would allow greater insights into the feeding efficiency and foraging patterns of whale sharks relative to their zooplankton prey.

### Supplementary Information

**Additional file 1**. Interactive 3D-UD of whale shark WS18_2018.

**Additional file 2**. Interactive 3D-UD of whale shark WS29_2018.

**Additional file 3**. Interactive 3D-UD of whale shark WS51_2018.

**Additional file 4**. Supplementary Figures.

## Data Availability

Data and developed code are available from the corresponding author upon request.

## Abbreviations

CTD: Conductivity, temperature, depth
VHF: Very high frequency
GPS: Global positioning system
TAD: Time at depth
TAT: Time at temperature
GPT: General purpose transceiver
LOPC: Laser optical plankton counter
GEBCO: General bathymetric charts of the oceans
NASC: Nautical area scattering coefficient
DWBA: Distorted wave born approximation
UD: Utilisation distribution
GLMM: Generalised linear mixed effects models
wAIC: Akaike’s information criterion weight
