# Supplementary figures and images for: Links between the three-dimensional movements of whale sharks (Rhincodon typus) and the bio-physical environment off a coral reef
Source: Mov Ecol. 2024 Jan 31;12:10. doi: 10.1186/s40462-024-00452-2 (PMC10829290; doi:10.1186/s40462-024-00452-2)

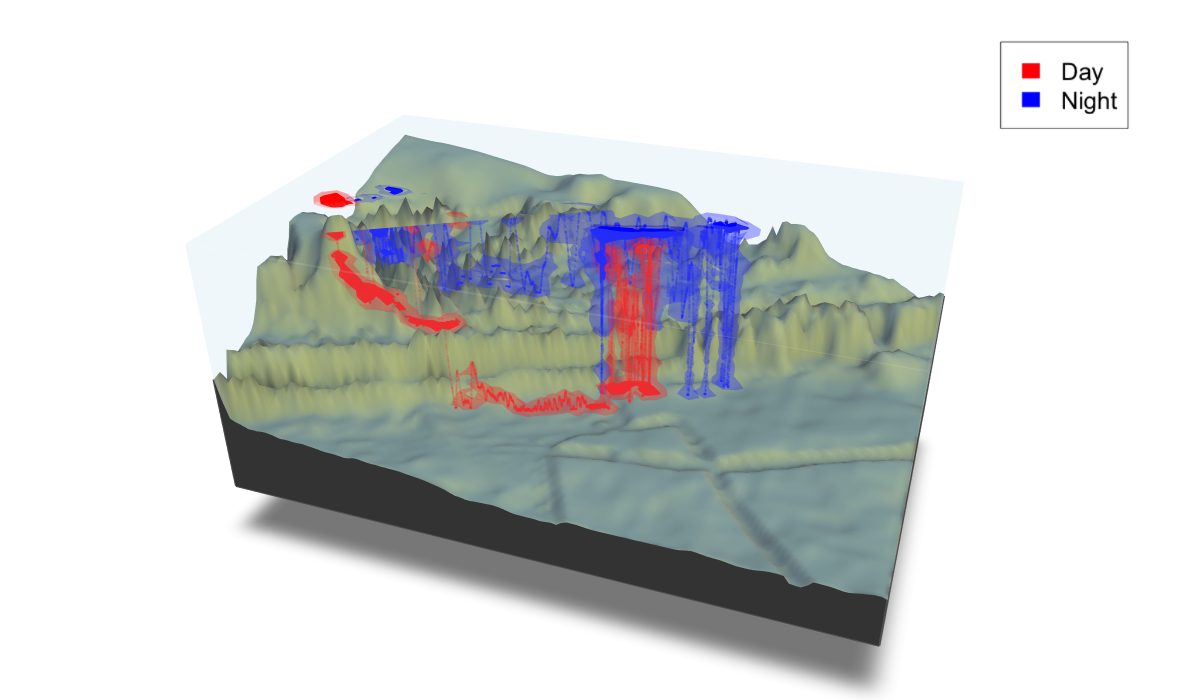

Supplement: Supplementary file 1 — Additional file 1. Interactive 3D-UD of whale shark WS18_2018. [file 40462_2024_452_MOESM1_ESM.zip › WS18_2018/WS18_2018.png]

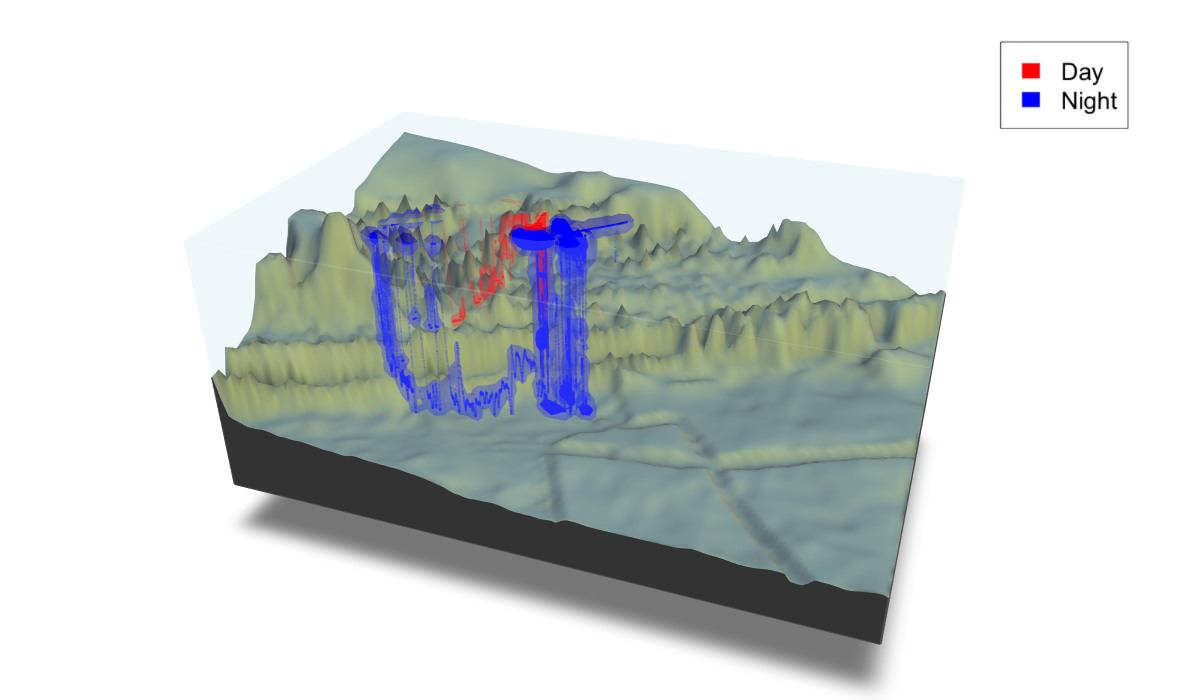

Supplement: Supplementary file 2 — Additional file 2. Interactive 3D-UD of whale shark WS29_2018. [file 40462_2024_452_MOESM2_ESM.zip › WS29_2018/WS29_2018.png]

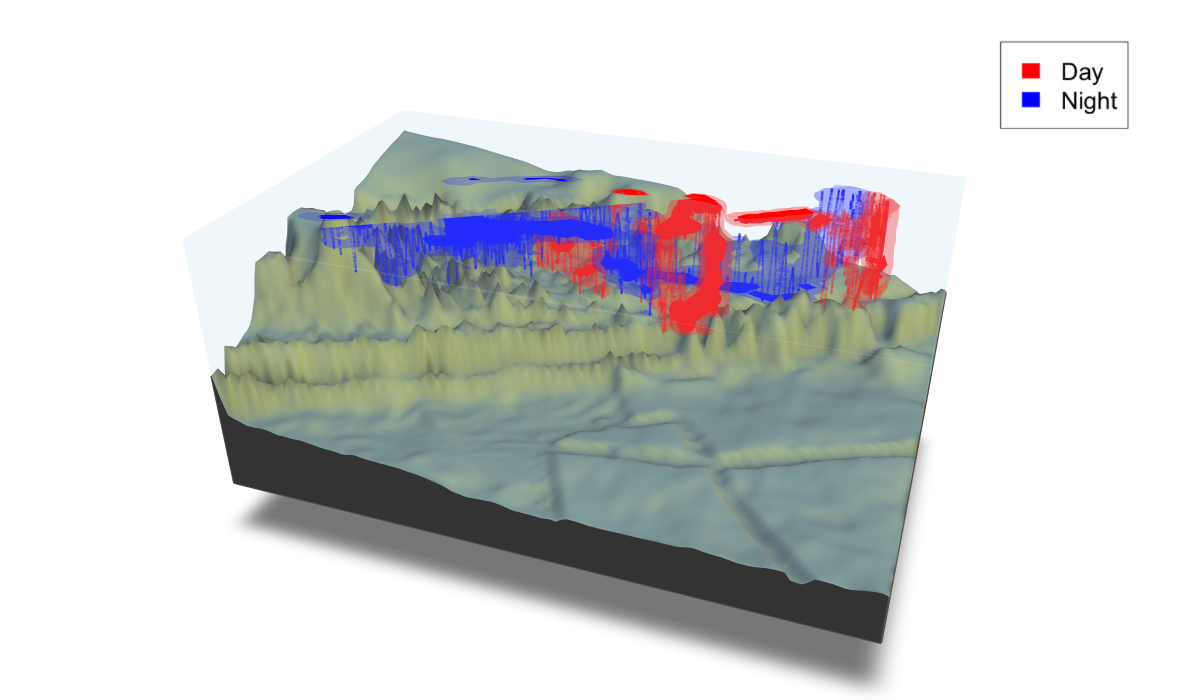

Supplement: Supplementary file 3 — Additional file 3. Interactive 3D-UD of whale shark WS51_2018. [file 40462_2024_452_MOESM3_ESM.zip › WS51_2018/WS51_2018.png]
