# Supplementary material for: Links between the three-dimensional movements of whale sharks (Rhincodon typus) and the bio-physical environment off a coral reef
Source: Mov Ecol. 2024 Jan 31;12:10. doi: 10.1186/s40462-024-00452-2 (PMC10829290; doi:10.1186/s40462-024-00452-2)
Supplement: Supplementary file 4 — Additional file 4. Supplementary Figures. [file 40462_2024_452_MOESM4_ESM.docx]

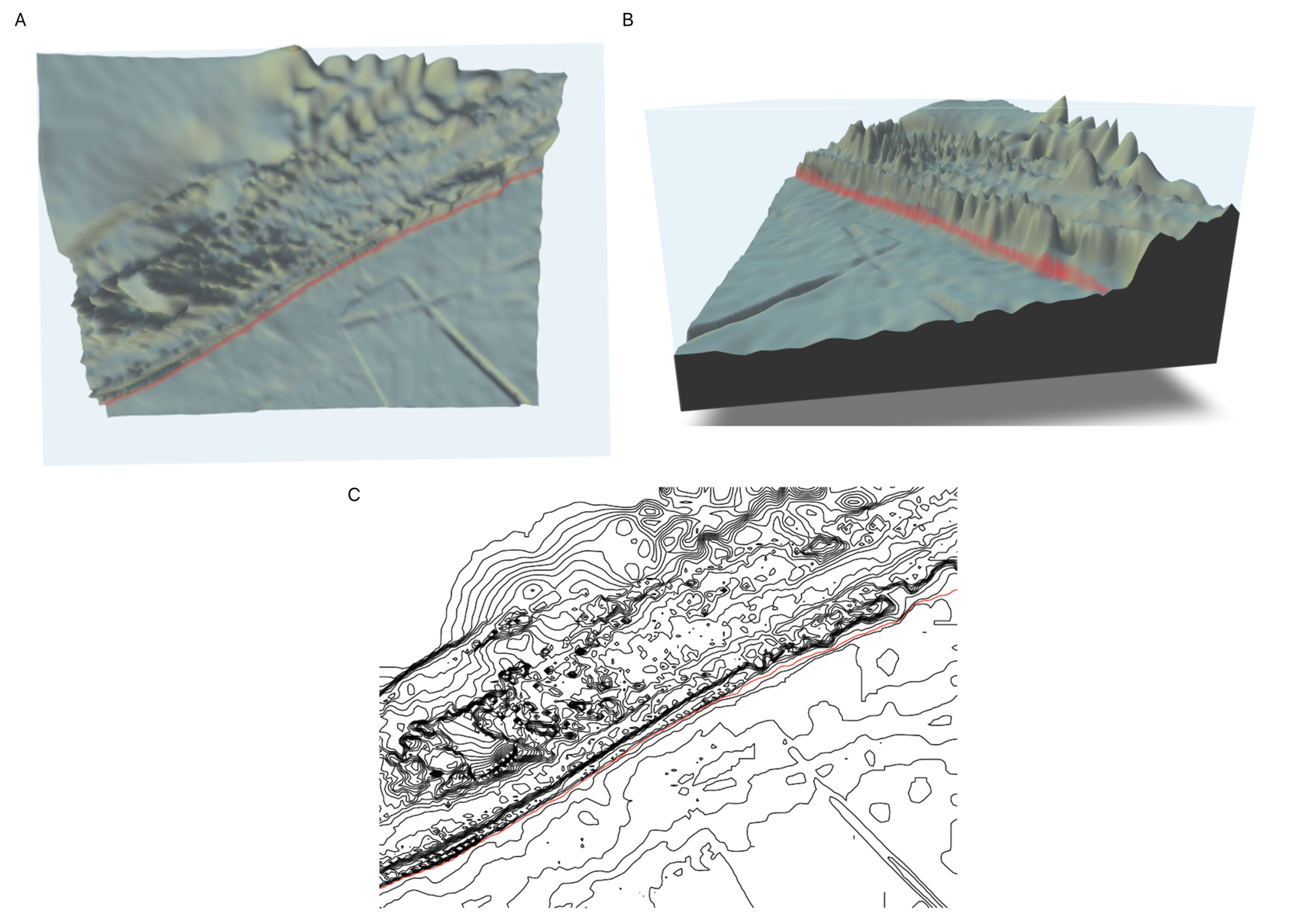


*Supplementary figure 1: The bathymetry data plotted in 3D (A & B) with the red line depicting the reef edge at the 50 m depth. (C) The bathymetry contours plotted in 2D with contour lines drawn at 2 m depth intervals and the reef edge at the 50 m depth contour depicted in red.*


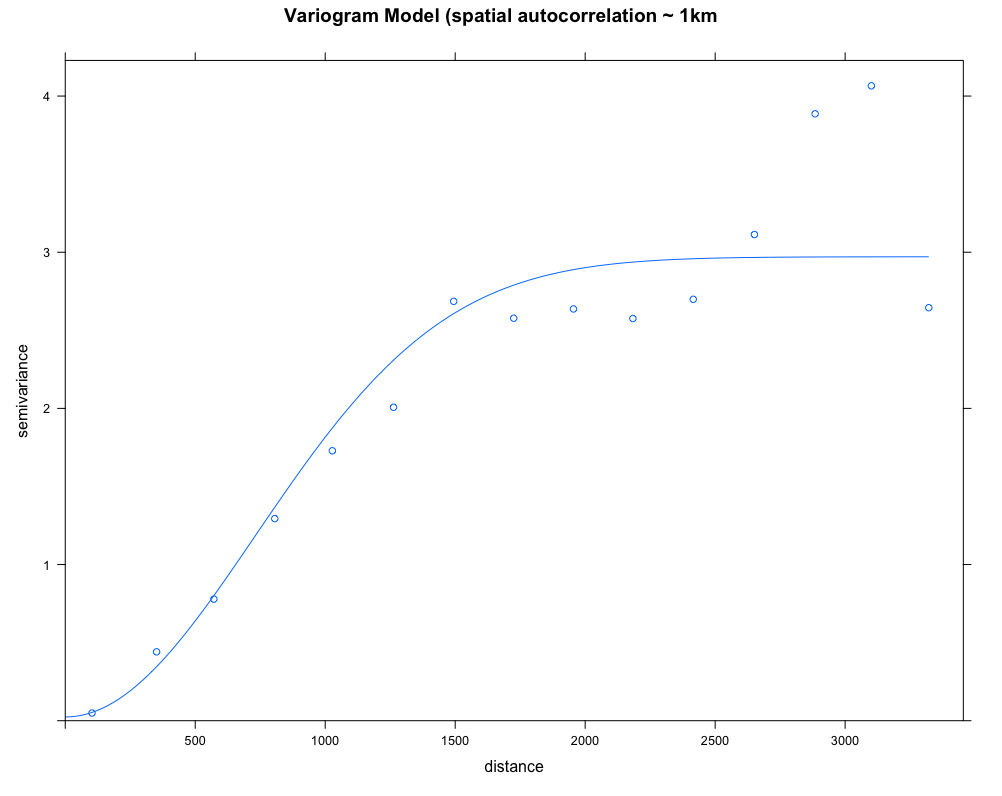


*Supplementary figure 2: A fitted variogram model with a gaussian distribution identifying spatial autocorrelation between points within the hydroacoustic transect ~ 1km apart.*

*
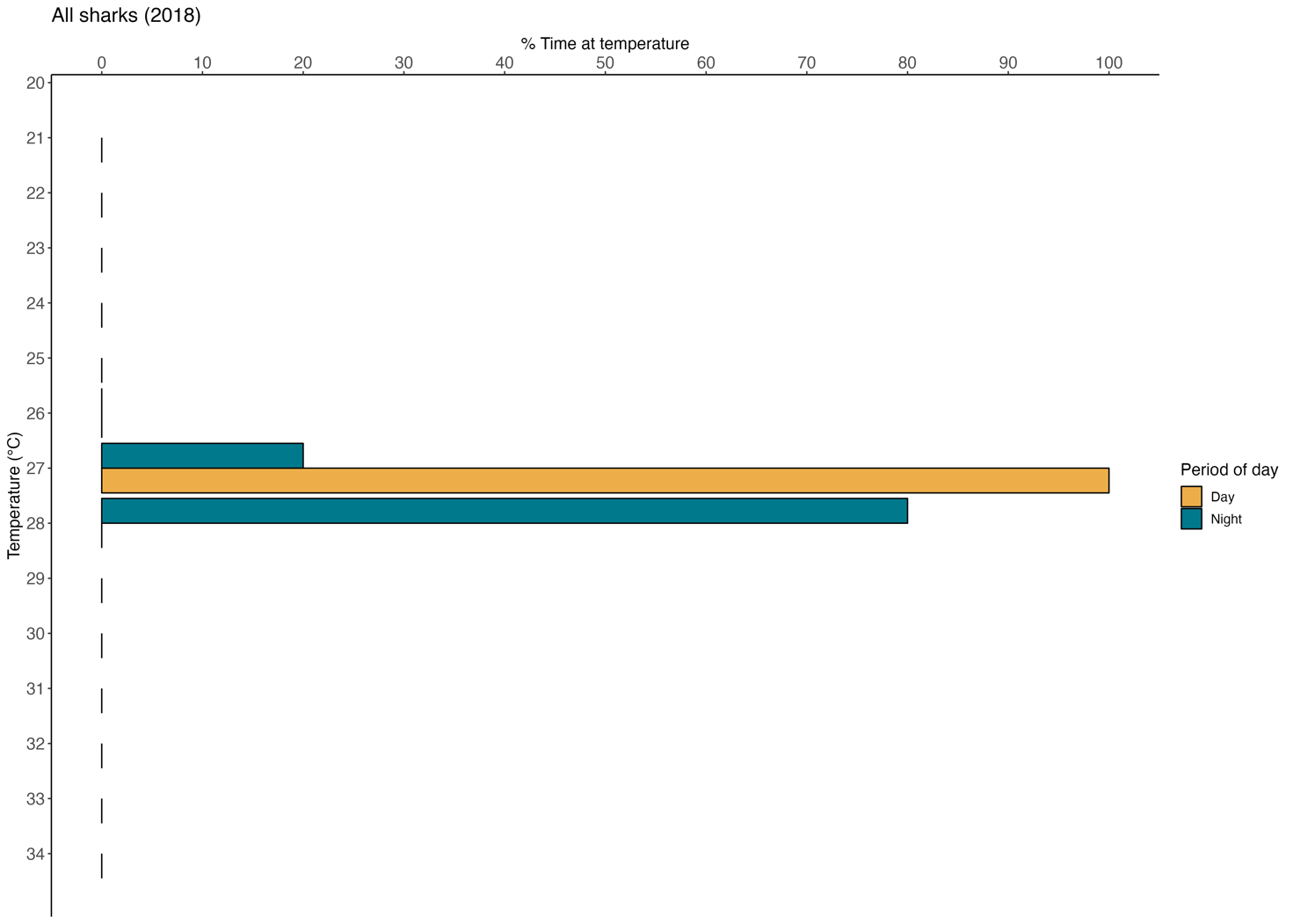
*

*Supplementary figure 3: Histograms of % of time sharks spent per temperature bin throughout the water column during both day and night hours for all tagged sharks.*


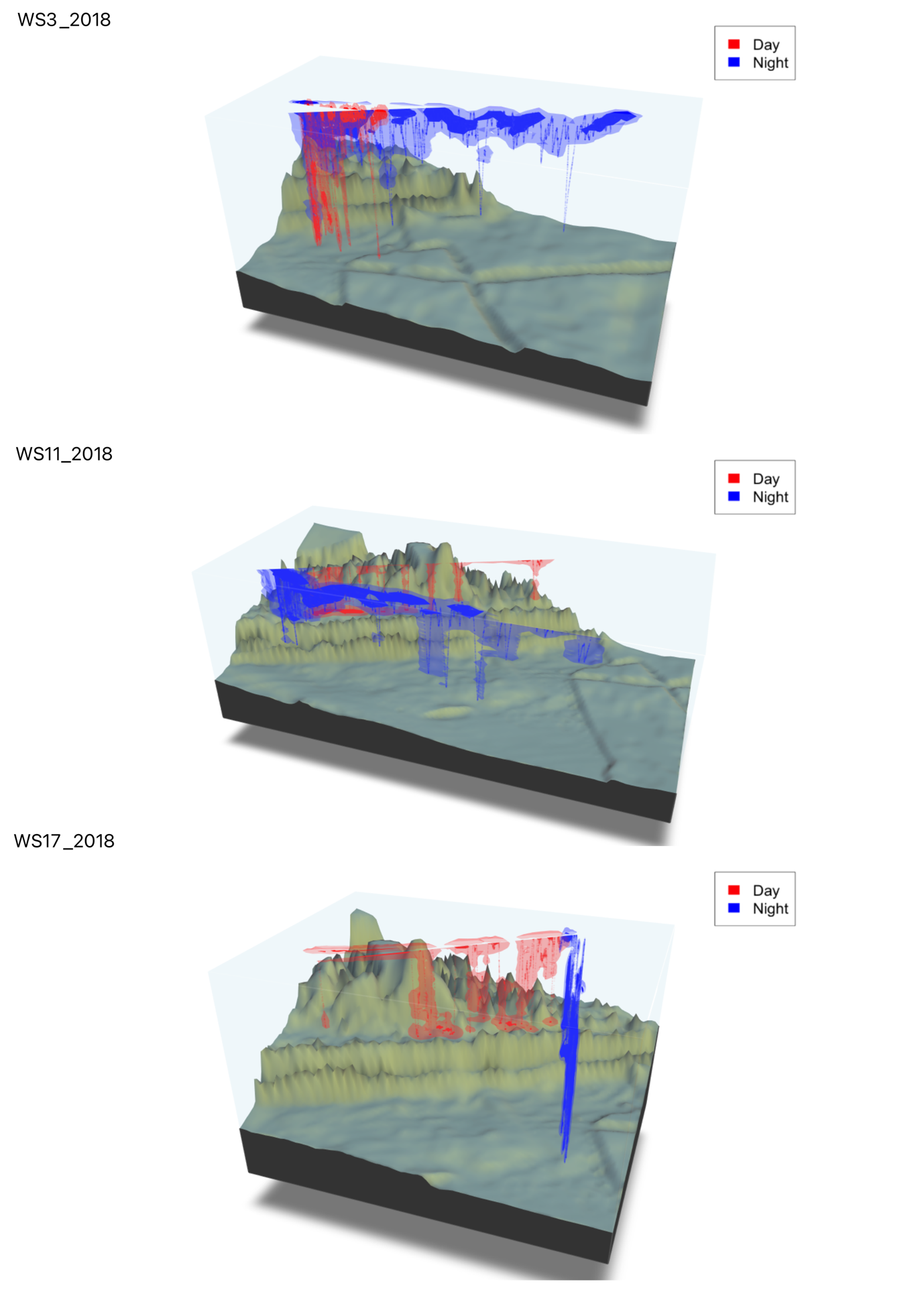


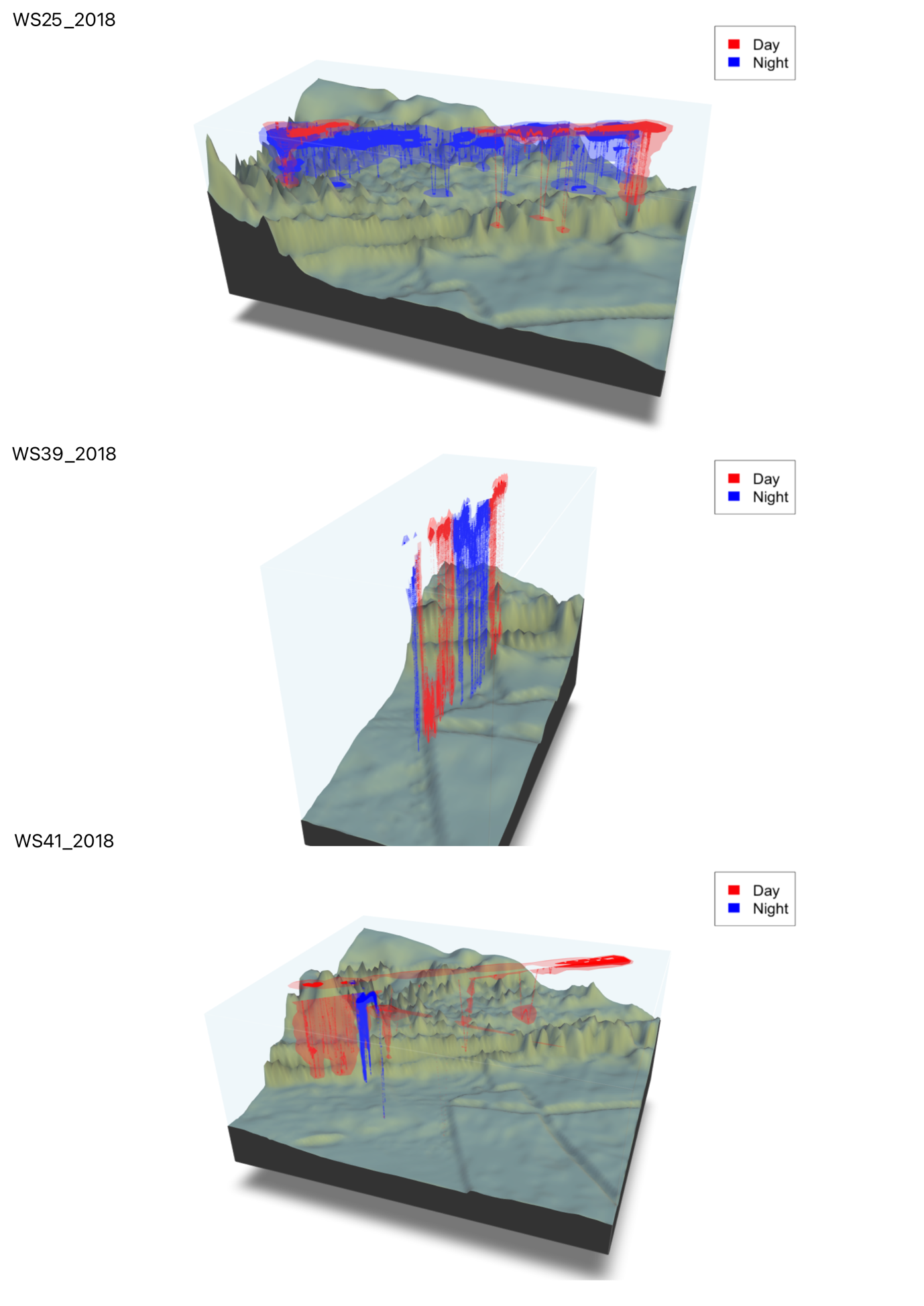


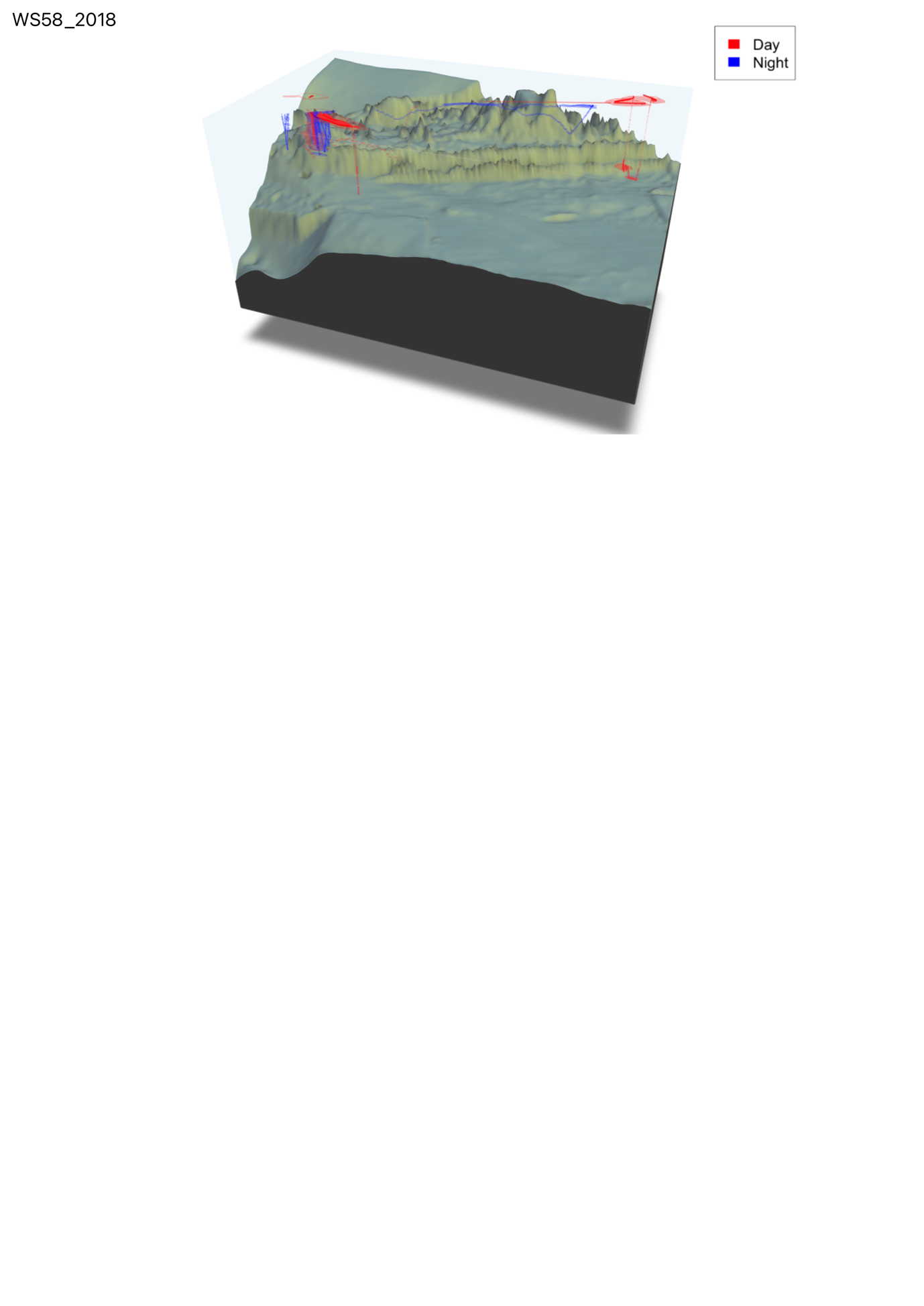


*Supplementary figure 4:* *The 50% (darker shades) and 95% (lighter shades) 3D UD space for all tracked whale sharks split by day and night in relation to the available bathymetry.*

*
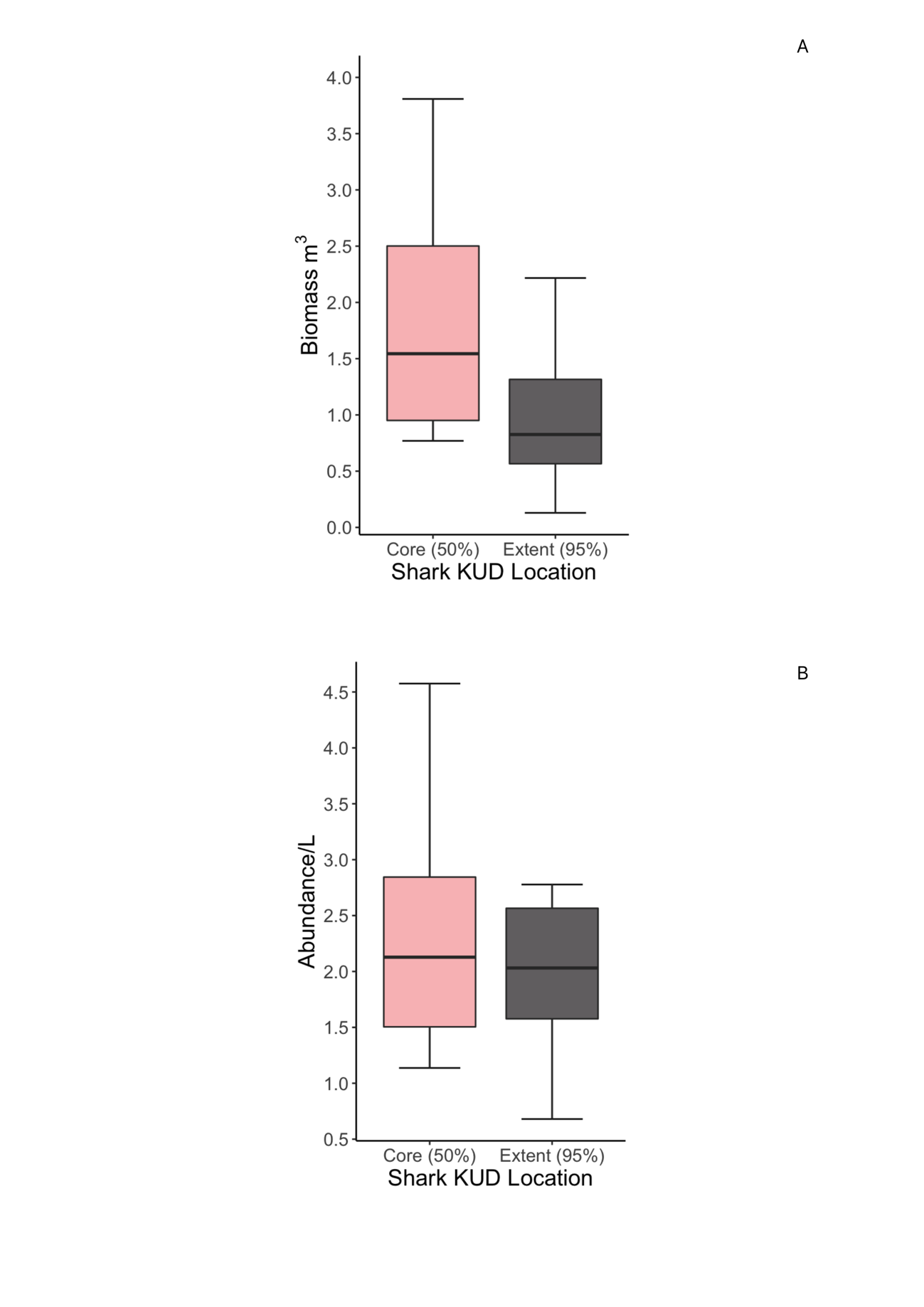
*

*Supplementary figure 5: The median zooplankton biomass m^3^ (A) and abundance/L (B) within the 50% UD (core) and 95% UD (extent) area of whale sharks.*

*
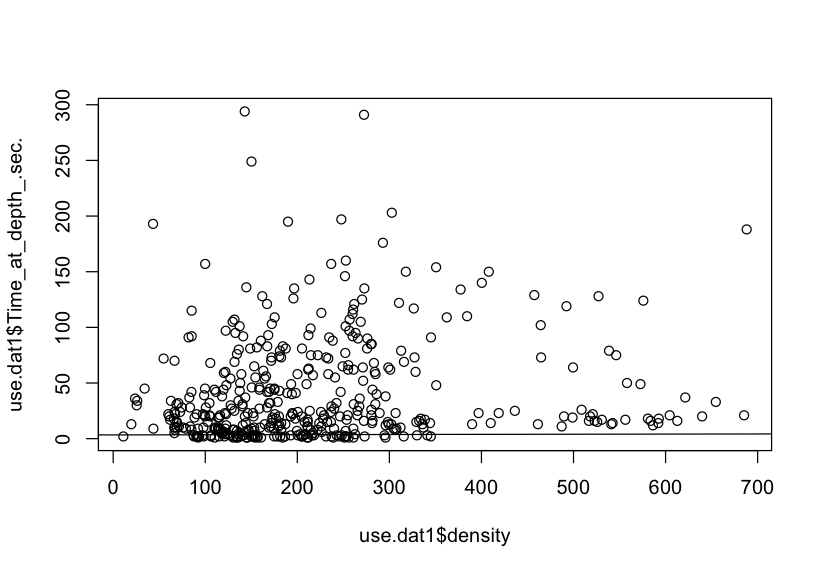
*

*Supplementary figure 6: A GLM model with prey density per m^3^ cropped between 5 and 50 meters as the predictor and shark TAD per 1 m depth bins as the response. Model reported an R^2^ value of 0.02.*

| *Supplementary table 1: Table describes the sex, size, latitude, longitude and date-time (AWST) for each whale shark observation our dataset.* | | | | | | | |
| --- | --- | --- | --- | --- | --- | --- | --- |
| **Shark observation  number** | **Latitude** | **Longitude** | **Sex** | **Length** | **Unique  Individual** | **Tag Deployment ID** | **Date-time  (AWST)** |
| 1 | -22.7630 | 113.6217 | F | 4 | Y |  | 15/05/2018 12:00 |
| 2 | -22.8186 | 113.6407 | M |  | Y |  | 15/05/2018 13:15 |
| 3 | -22.8132 | 113.6432 | M | 6 | Y | 168927 | 15/05/2018 13:35 |
| 4 | -22.7948 | 113.6443 | M | 3.5 | Y |  | 15/05/2018 14:15 |
| 5 | -22.7932 | 113.6410 | U | 6 | Y |  | 15/05/2018 14:25 |
| 6 | -22.7875 | 113.6385 | M | 7 | Y |  | 15/05/2018 14:40 |
| 7 | -22.7818 | 113.6392 | M | 7 | Y |  | 15/05/2018 15:00 |
| 8 | -22.7776 | 113.6371 | M | 3.5 |  |  | 15/05/2018 15:15 |
| 9 | -22.8321 | 113.6511 | M | 5 | Y |  | 16/05/2018 12:40 |
| 10 | -22.7956 | 113.6470 | M | 5 | Y |  | 16/05/2018 13:10 |
| 11 | -22.7946 | 113.6442 | M | 8 | Y | 168927 | 16/05/2018 13:30 |
| 12 | -22.7817 | 113.6363 | M | 5 |  |  | 16/05/2018 13:55 |
| 13 | -22.7799 | 113.6381 | U |  | Y |  | 16/05/2018 14:05 |
| 14 | -22.7458 | 113.6328 | F | 4.5 |  |  | 17/05/2018 14:20 |
| 15 | -22.7739 | 113.6399 | M | 3.5 | Y |  | 17/05/2018 15:10 |
| 16 | -22.7912 | 113.6495 | U | 7 | Y |  | 17/05/2018 15:20 |
| 17 | -22.7961 | 113.6569 | M | 7 |  | 168930 | 17/05/2018 15:50 |
| 18 | -22.7859 | 113.6487 | F | 7 | Y | 168927 | 18/05/2018 13:00 |
| 19 | -22.7940 | 113.6526 | M | 5 |  |  | 18/05/2018 13:15 |
| 20 | -22.7974 | 113.6525 | M | 5 | Y |  | 18/05/2018 13:20 |
| 21 | -22.7809 | 113.6411 | M | 8 |  |  | 18/05/2018 13:45 |
| 22 | -22.7889 | 113.6482 | M | 7 |  |  | 18/05/2018 13:55 |
| 23 | -22.7774 | 113.6509 | U |  |  |  | 18/05/2018 15:00 |
| 24 | -22.8098 | 113.6669 | F | 5 |  |  | 19/05/2018 11:25 |
| 25 | -22.8238 | 113.6699 | M | 6 | Y | 168928 | 19/05/2018 11:35 |
| 26 | -22.8021 | 113.6614 | F | 5 | Y |  | 19/05/2018 12:05 |
| 27 | -22.8369 | 113.6621 | M | 6 |  |  | 19/05/2018 12:20 |
| 28 | -22.8162 | 113.6570 | F |  |  |  | 19/05/2018 12:30 |
| 29 | -22.8030 | 113.6540 | M | 6 | Y | 168930 | 19/05/2018 12:40 |
| 30 | -22.8147 | 113.6465 | F | 5 |  |  | 19/05/2018 13:00 |
| 31 | -22.8127 | 113.6426 | F | 5 |  |  | 19/05/2018 13:15 |
| 32 | -22.8105 | 113.6456 | F | 4.5 |  |  | 19/05/2018 13:20 |
| 33 | -22.8163 | 113.6635 | M | 6 |  |  | 19/05/2018 13:55 |
| 34 | -22.8123 | 113.6342 | F | 4.5 |  |  | 19/05/2018 14:15 |
| 35 | -22.7738 | 113.6419 | M | 4 |  |  | 19/05/2018 14:20 |
| 36 | -22.7740 | 113.6436 | M | 7 | Y |  | 19/05/2018 15:20 |
| 37 | -22.8144 | 113.6564 | F | 5 |  |  | 19/05/2018 15:50 |
| 38 | -22.8143 | 113.6563 | U |  | Y |  | 20/05/2018 11:55 |
| 39 | -22.8133 | 113.6604 | M |  | Y |  | 20/05/2018 12:00 |
| 40 | -22.8309 | 113.6747 | M | 5 | Y |  | 20/05/2018 12:25 |
| 41 | -22.8342 | 113.6705 | M | 6 | Y | 168927 | 20/05/2018 13:35 |
| 42 | -22.8229 | 113.6661 | M | 5 |  |  | 20/05/2018 13:50 |
| 43 | -22.8404 | 113.6886 | M | 5 |  |  | 20/05/2018 14:05 |
| 44 | -22.8442 | 113.6801 | M | 6 | Y |  | 20/05/2018 14:25 |
| 45 | -22.8516 | 113.6769 | M | 4 |  |  | 20/05/2018 14:55 |
| 46 | -22.8268 | 113.6711 | M | 6 |  |  | 20/05/2018 15:10 |
| 47 | -22.8172 | 113.6823 | M | 3 | Y |  | 20/05/2018 15:20 |
| 48 | -22.8232 | 113.6516 | M | 4 | Y |  | 21/05/2018 11:50 |
| 49 | -22.8275 | 113.6659 | M |  |  |  | 21/05/2018 12:30 |
| 50 | -22.8759 | 113.7011 | F | 4 |  |  | 21/05/2018 13:00 |
| 51 | -22.8051 | 113.6765 | M | 8 |  | 168930 | 21/05/2018 14:10 |
| 52 | -22.8039 | 113.6711 | U | 5 |  |  | 21/05/2018 14:20 |
| 53 | -22.7901 | 113.6718 | M | 6 |  |  | 21/05/2018 14:30 |
| 54 | -22.7964 | 113.6567 | M | 4.5 | Y |  | 21/05/2018 14:40 |
| 55 | -22.7903 | 113.6366 | M | 5 |  |  | 21/05/2018 14:50 |
| 56 | -22.7779 | 113.6028 | U |  |  |  | 21/05/2018 15:20 |
| 57 | -22.7965 | 113.6567 | M | 6 | Y |  | 22/05/2018 12:40 |
| 58 | -22.7976 | 113.6658 | M | 6 | Y | 168927 | 22/05/2018 13:00 |
| 59 | -22.7983 | 113.6682 | M | 7 |  |  | 22/05/2018 13:10 |
| 60 | -22.8313 | 113.6798 | M | 3.5 | Y |  | 22/05/2018 13:35 |
| 61 | -22.8146 | 113.6745 | M | 6 |  |  | 22/05/2018 13:47 |
| 62 | -22.8003 | 113.6685 | U | 6 | Y |  | 22/05/2018 14:10 |
| 63 | -22.8019 | 113.6719 | F | 6 |  |  | 22/05/2018 14:20 |
| 64 | -22.8019 | 113.6718 | M | 6 |  |  | 22/05/2018 14:20 |
| 65 | -22.7942 | 113.6785 | M | 6 | Y |  | 22/05/2018 14:40 |
| 66 | -22.8180 | 113.6609 | M | 6 |  |  | 22/05/2018 15:10 |
| 67 | -22.8111 | 113.6678 | U |  | Y |  | 22/05/2018 15:30 |
| 68 | -22.8089 | 113.6683 | M | 5 | Y |  | 22/05/2018 15:55 |
| 69 | -22.8089 | 113.6684 | M | 9 | Y |  | 22/05/2018 16:00 |
| 70 | -22.8043 | 113.6659 | M | 8 | Y |  | 22/05/2018 16:30 |
| 71 | -22.8006 | 113.6874 | U | 6 |  |  | 23/05/2018 11:25 |
| 72 | -22.7798 | 113.6421 | M | 6 | Y |  | 23/05/2018 13:00 |
| 73 | -22.7894 | 113.6554 | M | 8 | Y |  | 23/05/2018 13:25 |
| 74 | -22.8085 | 113.6715 | U | 4.5 |  |  | 23/05/2018 13:35 |
| 75 | -22.8133 | 113.6653 | F | 4.5 |  |  | 23/05/2018 14:00 |
| 76 | -22.8139 | 113.6772 | M | 6 |  |  | 23/05/2018 14:15 |
| 77 | -22.8131 | 113.6754 | M | 6 |  |  | 23/05/2018 14:25 |
| 78 | -22.8077 | 113.6587 | U | 4.5 | Y |  | 23/05/2018 15:00 |
| 79 | -22.7987 | 113.6621 | M | 8 | Y |  | 23/05/2018 15:30 |
